# ASXL1 in proinflammatory macrophages attenuates inflammatory activation and macrophage‐mediated renal tubular epithelial cell damage in acute kidney injury

**DOI:** 10.14814/phy2.71119

**Published:** 2026-09-30

**Authors:** Yoshiyasu Ogura, Takehito Sugasawa, Masaomi Nangaku, Imari Mimura

**Affiliations:** ^1^ Division of Nephrology and Endocrinology The University of Tokyo Graduate School of Medicine Tokyo Japan; ^2^ Department of Clinical Medicine, Faculty of Medicine, and the Department of Sports Medicine Analysis, Research Facility Center for Science and Technology University of Tsukuba Ibaraki Japan; ^3^ Organ Pathophysiology Program The University of Tokyo Graduate School of Medicine Tokyo Japan

**Keywords:** AKI, ASXL1, inflammation, macrophages

## Abstract

Macrophages are major contributors to inflammatory responses in acute kidney injury (AKI) and rapidly alter their gene expression through epigenetic regulation. This study focused on ASXL1, a chromatin modifier regulating histone marks consisting of H3K27me3 (repressive) and H3K4me3 (activating) in M1 macrophages. We found that ASXL1‐positive M1 macrophages were significantly increased in the unilateral ischemia–reperfusion injury kidneys of male mice in the acute phase. In cultured M1 macrophages, RNA sequencing revealed that *Asxl1* knockdown upregulated inflammatory response genes such as *Il6* and *Il1b*. Conversely, the expression of anti‐apoptotic genes *Csf3* and *Lcn2* was suppressed, and pathways related to interleukin‐17 signaling and Fcγ receptor‐dependent phagocytosis were downregulated. In co‐culture experiments, *Asxl1* silencing in M1 macrophages induced more severe apoptosis and reduced proliferation of mouse renal cortical tubular cells. Mechanistically, chromatin immunoprecipitation analysis demonstrated that *Asxl1* knockdown significantly decreased H3K27me3 levels at the *Il6* and *Il1b* promoter regions and H3K4me3 levels at the *Csf3* and *Lcn2* promoter regions in M1 macrophages. These findings indicate that ASXL1 suppresses excessive inflammatory cytokine expression and preserves anti‐apoptotic activity through histone modifications, while maintaining phagocytic capacity in M1 macrophages. ASXL1 in M1 macrophages thus attenuates inflammatory activation and macrophage‐mediated renal tubular cell damage in AKI.

## Graphical abstract

ASXL1 in inflammatory macrophages attenuates renal tubular cell damage through coordinated histone modification of H3K27me3 and H3K4me3 in acute kidney injury.

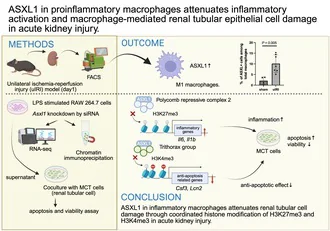

## 1 INTRODUCTION

Acute kidney injury (AKI) is a growing global health concern and a common clinical condition characterized by a sudden decline in kidney function. AKI is associated not only with substantial morbidity and mortality but also with an increased risk of developing chronic kidney disease (Coca et al., 2012). Recent studies have shown that both innate and adaptive immune responses play key roles in mediating renal tubular cell damage and promoting recovery following AKI. Dendritic cells, monocytes, macrophages, neutrophils, T lymphocytes, and B lymphocytes all contribute to kidney injury (Bonavia & Singbartl, 2018). Among these immune cells, macrophages are particularly multifunctional. Beyond their phagocytic activity, they perform a wide range of complex functions in host defense, tissue development, and homeostasis, as well as in tissue injury, repair, and fibrosis (Wynn & Vannella, 2016).

Tissue macrophages are highly responsive to environmental stimuli and can adopt a classically activated, proinflammatory M1 phenotype (Martinez et al., 2008). These M1 macrophages are typically activated by lipopolysaccharides (LPS), the ligands of Toll‐like receptors (TLRs), or by interferon gamma. Upon activation, they express high levels of inducible nitric oxide synthase and inflammatory cytokines such as tumor necrosis factor, interleukin (IL)‐6, IL‐1β, and IL‐12 (Huen & Cantley, 2017). In the kidney, inflammation primarily driven by M1 macrophages represents a key hallmark of the response in AKI (Han et al., 2019; Huen & Cantley, 2017). This M1‐mediated inflammatory process leads to increased oxidative damage, endothelial dysfunction, and cell death through apoptosis and necrosis, accompanied by immune cell accumulation, ultimately resulting in impaired kidney function (Bonventre & Yang, 2011; Ferenbach et al., 2012; Lee et al., 2011; Lu et al., 2012).

Macrophages are among the most transcriptionally dynamic cells, capable of rapidly altering their transcriptional output to adapt to environmental changes during infection or tissue injury (Gosselin & Glass, 2014). Epigenetic regulation, closely linked to the structural and spatial organization of the genome, governs global gene expression patterns in macrophages (Gosselin & Glass, 2014). The most well‐recognized epigenetic mechanisms include DNA methylation, histone modifications, and non‐coding RNAs (Li, 2021). These modifications influence the accessibility of transcription factor binding sites, thereby regulating gene transcription and cellular function (Peixoto et al., 2020). In macrophages, histone modifications are key regulators of phenotype determination (Wang et al., 2024). For example, specific enzymes that regulate histone methylation and acetylation, such as Kdm6a and histone deacetylase 3 (HDAC3), directly control macrophage inflammatory responses and M1 polarization (Ghiboub et al., 2020; Karnam et al., 2023; Kouzarides, 2007; Li et al., 2017).

In the context of macrophage‐mediated kidney injury, clonal hematopoiesis of indeterminate potential (CHIP) has recently garnered significant attention regarding its role in the epigenetic regulation of macrophage inflammatory phenotypes (Kestenbaum et al., 2023; Ogura & Mimura, 2023; Vlasschaert et al., 2022). Genome‐wide analyses have revealed that the accumulation of somatic mutations in hematopoietic stem cells increases with aging. This leads to clonal expansion, referred to as CHIP, which represents a premalignant state associated with hematologic cancers (Steensma et al., 2015). The most frequently mutated genes in CHIP are epigenetic regulators, including *DNMT3A* and *Tet2*, which encode DNA methylation enzymes, and *Asxl1*, a chromatin regulator (Steensma et al., 2015). Hematopoietic stem cells carrying CHIP‐driving variants, particularly those with myeloid‐specific mutations, exhibit a shift toward increased myeloid progenitor production and are associated with enhanced proinflammatory macrophage infiltration in various organs (Jaiswal, 2020). CHIP promotes systemic inflammation and has been linked not only to hematologic malignancies but also to non‐malignant conditions such as atherosclerosis, cardiovascular disease, and kidney injury (Jaiswal, 2020; Jaiswal et al., 2017; Vlasschaert et al., 2024). In population‐based epidemiologic cohorts, meta‐analyses have shown that non‐*DNMT3A* CHIP, including *Asxl1*‐related CHIP, is associated with a higher risk of incident AKI and impaired recovery from it (Vlasschaert et al., 2024). Moreover, studies using *Tet2*‐related CHIP mouse models of AKI demonstrated increased renal proinflammatory macrophage infiltration and greater post‐AKI kidney fibrosis (Vlasschaert et al., 2024).

The additional sex comb‐like (ASXL) family of proteins comprises chromatin‐associated factors involved in both transcriptional activation and repression (Milne et al., 1999). Originally identified in *Drosophila* as enhancers of the Trithorax group (TrxG) and Polycomb group genes, ASXL proteins play a crucial role in regulating *Hox* gene expression (Milne et al., 1999; Sinclair et al., 1998). The TrxG complex catalyzes the trimethylation of histone H3 at lysine 4 (H3K4me3), leading to transcriptional activation (Schuettengruber et al., 2017), whereas the Polycomb repressive complex 2 (PRC2) trimethylates histone H3 at lysine 27 (H3K27me3), thereby repressing target gene expression (Schuettengruber et al., 2017). Mutations in *ASXL1* are frequently detected in various myeloid malignancies (Carbuccia et al., 2009) and in Bohring‐Opitz syndrome (Hoischen et al., 2011). ASXL1 is essential for normal hematopoiesis, and the wild‐type protein epigenetically regulates gene expression by maintaining appropriate levels of H3K4me3 and H3K27me3 (Asada et al., 2019).

To our knowledge, no study has yet examined the role of ASXL1 in macrophages that accumulate within injured kidneys. Given clinical evidence linking *Asxl1*‐related CHIP to an increased risk of AKI (hazard ratio, 1.36; 95% confidence interval, 1.17–1.59) (Vlasschaert et al., 2024), ASXL1 may influence inflammatory regulation in macrophages through histone modifications such as H3K4me3 and H3K27me3 during AKI. However, the specific role of ASXL1 in macrophages within injured kidneys remains unclear. We hypothesized that ASXL1 contributes to the regulation of the inflammatory phenotype of macrophages in AKI. To test this hypothesis, we investigated the role of ASXL1 in inflammatory macrophages during AKI, with particular focus on its function in M1 polarization (LPS‐induced inflammatory macrophages).

## 2 MATERIALS AND METHODS

### 2.1 Animal model

Ten‐ to twelve‐week‐old male mice were used in this study. C57BL/6 mice were purchased from CLEA Japan, Inc. (Tokyo, Japan). All mice were maintained under standard conditions (25°C temperature, 50% humidity, and 12‐h light/dark cycle), with a standard laboratory diet (MF; Oriental Yeast Co., Ltd., Tokyo, Japan) and water available ad libitum. After 1 week of acclimatization, the mice underwent unilateral ischemia–reperfusion injury (uIRI). Under anesthesia induced by intraperitoneal injection of midazolam (Sandoz, Tokyo, Japan), butorphanol (Meiji Seika Pharma Co., Ltd., Tokyo, Japan), and medetomidine (Kyoritsu Seiyaku, Tokyo, Japan), the left renal pedicle was clamped for 30 min and then reperfused (*n* = 6). Sham‐operated mice were anesthetized in the same manner but underwent only laparotomy without clamping (*n* = 4). All mice were euthanized 24 h after the procedure by exsanguination under deep intraperitoneal anesthesia, and the left kidneys were collected for analysis. All animal experiments were approved by the Graduate School of Medicine, University of Tokyo (approval no. A23M0157) and conducted in accordance with the guidelines established by the Committee on Ethical Animal Care and Use at the University of Tokyo.

### 2.2 Immunostaining

Kidneys were fixed in Mildform 10N (133‐103111; Wako, Osaka, Japan) and processed for histological analysis using periodic acid–Schiff and hematoxylin and eosin staining or embedded in paraffin for immunostaining (3‐μm sections). For immunostaining, 3‐μm paraffin sections of mice kidneys were deparaffinized and subjected to antigen retrieval by boiling in citrate buffer (pH 6.0). For fluorescent staining, kidneys were frozen in OCT compound. 8‐μm kidney sections were cut using a freezing microtome, fixed with 4% paraformaldehyde (PFA), and permeabilized with 0.3% Triton X‐100. Sections were then blocked at room temperature using Protein Block Serum‐Free (X0909; Agilent, Santa Clara, CA). The following primary antibodies were used: anti CD68 (E3O7V; 1:400, #97778; Cell Signaling Technology, Danvers, MA), anti‐ASXL1 (1:50, PA5‐71475; Thermo Fisher Scientific, Waltham, MA), anti‐KIM‐1 (1:2000, AF1817; R&D Systems, Minneapolis, MN). For immunohistochemistry, staining was performed using the Histofine Simple Stain Mouse MAX PO (R) kit (414,341; Nichirei, Tokyo, Japan) and ImmPACT DAB substrate (SK‐4105; Vector Laboratories, Burlingame, CA). For immunofluorescence, the following secondary antibodies were used: donkey anti‐rabbit immunoglobulin G (IgG; H + L) Alexa Fluor™ 488 (1:1000, A‐21206; Thermo Fisher Scientific, Waltham, MA), donkey anti‐rat IgG (H&L) Alexa Fluor™ 594 (1:1000, A‐21209; Thermo Fisher Scientific, Waltham, MA), donkey anti‐goat IgG (H&L) Alexa Fluor® 594 (1:1000, 705‐585‐147; Jackson Immuno Research, West Grove, PA), and donkey anti‐mouse IgG (H&L) Alexa Fluor® 594 (1:1000, ab150108; Abcam, Cambridge, UK). Quantification of marker‐positive cells was performed in a blinded manner by counting positive cells in 10 randomly selected cortical fields at 400× magnification. Images were captured at 400× or 1000× magnification using a KEYENCE BZ‐X710 microscopy system.

The immunofluorescence co‐staining of ASXL1 and CD68 was evaluated as follows: ASXL1 was stained green (Alexa 488) in the nucleus and cytoplasm, CD68 was stained red (Alexa 594) in the cytoplasm, and nuclei were stained blue (Hoechst). When all three were stained in the same cell, it was defined as an ASXL1‐positive macrophage, and when ASXL1 was not stained, it was defined as an ASXL1‐negative macrophage. In the figure, the former are indicated by white arrows, and the latter by white arrowheads (Figure 1i).

### 2.3 Flow cytometry

Single‐cell suspensions were prepared from mice kidneys using the Muti Tissue Dissociation Kit 2 (# 130‐110‐203; Miltenyi Biotec, North Rhine‐Westphalia, Germany). Representative tissue sections from uIR or sham kidneys were used for flow cytometry. Tissues were weighed and digested using gentleMACS Octo Dissociator with Heaters (# 130‐096‐427; Miltenyi Biotec, North Rhine‐Westphalia, Germany) to obtain a single‐cell suspension. Cells were fixed and permeabilized using Transcription Factor Staining Buffer Set (130‐122‐981; Miltenyi Biotec, North Rhine‐Westphalia, Germany), then incubated with staining mix on ice (Viobility 405/520 Fixable Dye (130–130‐404), 1:1000 from Miltenyi Biotec (North Rhine‐Westphalia, Germany); CD45‐PB (103125), 1:2000, CD11b‐APC (101212), 1:800, Ly‐6G‐PerCP (127653), 1:1000, F4/80‐APC/Cy7 (123117), 1:500 from BioLegend (San Diego, CA), ASXL1‐FITC (custom‐conjugated; Thermo Fisher Scientific, Waltham, MA), 1:500 and CD86‐PE (12‐0862‐81), 1:1400 from Invitrogen (San Diego, CA)) and analyzed on MACSQuant Analyzer 10 (Miltenyi Biotec, North Rhine‐Westphalia, Germany). After live CD45+ events were gated, myeloid cell populations were assessed using the markers as depicted in Figure S1. Absolute cell count was calculated using the weight of renal tissue in relation to event counts on MACSQuant Analyzer 10.

### 2.4 Cell culture and treatment

RAW 264.7 cells (a mouse macrophage cell line) were cultured in high‐glucose Dulbecco's Modified Eagle Medium (D5796; Sigma‐Aldrich, St. Louis, MO) supplemented with 10% fetal bovine serum (F7524m kit #BCBT 3830; Sigma‐Aldrich, St. Louis, MO). MCT cells (a mouse renal cortical tubular epithelial cell line) were cultured in low‐glucose Dulbecco's Modified Eagle Medium (D6046; Sigma‐Aldrich, St. Louis, MO) containing 5% fetal bovine serum and 1% antibiotics. All cells were maintained in a humidified atmosphere of 5% CO_2_ at 37°C. RAW 264.7 cells were treated with LPS (100 ng/mL; tlrl‐3pelps; InvivoGen, San Diego, CA) for 24 h to induce M1‐type macrophage polarization.

### 2.5 Oligonucleotide transfection

RAW 264.7 cells (1.0 × 10^5^ cells/well) were seeded in six‐well plates (140,675; Thermo Fisher Scientific, Waltham, MA) and cultured in standard growth medium. Small interfering RNA (siRNA) transfection was performed using Opti‐MEM I Reduced Serum Medium (31985070; Thermo Fisher Scientific, Waltham, MA) and Lipofectamine RNAiMAX Transfection Reagent (13778150; Thermo Fisher Scientific, Waltham, MA) according to the instructions of the manufacturer. ASXL1 knockdown was achieved using ON‐TARGETplus Mouse ASXL1 SMARTpool siRNA (#L‐046553‐01‐0005228790; Horizon Discovery, Lafayette, CO). ON‐TARGETplus Non‐targeting Pool siRNA (#D‐001810‐10‐05; Horizon Discovery, Lafayette, CO) served as the negative control. The final siRNA concentration was 20 nM. After 24 h of siRNA transfection, the medium was replaced with standard culture medium. The cells were then treated with LPS (100 ng/mL) or vehicle (UltraPure DNase/RNase‐Free Distilled Water, 10977015; Thermo Fisher Scientific, Waltham, MA) for 24 h. Following treatment, cells were collected for quantitative real‐time polymerase chain reaction (PCR) and chromatin immunoprecipitation analyses, while culture supernatants were used for enzyme‐linked immunosorbent assay (ELISA) measurement of IL‐6.

Overexpression by plasmid DNA electroporation.

ASXL1 expression plasmid constructed in the pcDNA3.1(+) plasmid was purchased from Vector Builder (Yokohama, Japan). pcDNA™3.1(+) mammalian expression vector (V79020; Invitrogen, San Diego, CA) was used as control empty (mock) plasmid. Since high concentrations of plasmids are required for the plasmid transfection into mammalian cell lines, plasmid preparation was performed using PureYield™ Plasmid Midiprep System (A2492; Promega, Madison, WI). RAW264.7 cells were electroporated with ASXL1 expression plasmid or mock pcDNA3.1(+) vectors via Neon™ Transfection System (Invitrogen, San Diego, CA) according to the manufacturer's protocol (pulse voltage: 1380 V, pulse width: 20 ms, pulse number: 1, cell density: 3.0 × 10^7^ cells/mL) and were seeded in 12‐well plates and cultured. For each transfection, 1 μg of plasmid DNA was used. The cells were then treated with LPS (100 ng/mL) or vehicle (UltraPure DNase/RNase‐Free Distilled Water, 10977015; Thermo Fisher Scientific, Waltham, MA) for 24 h. Following treatment, cells were collected for quantitative real‐time polymerase chain reaction (PCR), while culture supernatants were used for enzyme‐linked immunosorbent assay (ELISA) measurement of IL‐6.

### 2.6 Analysis of messenger RNA (mRNA)

Total RNA was extracted using RNAiso Plus (9108; Takara, Shiga, Japan) and reverse‐transcribed into complementary DNA with the PrimeScript RT Master Mix (RR036B; Takara, Shiga, Japan). Quantitative real‐time PCR was performed using the THUNDERBIRD qPCR Mix (QPS‐101; Toyobo, Tokyo, Japan) on a CFX96 Real‐Time System (Bio‐Rad Laboratories, Inc., Hercules, CA). β‐actin served as internal controls for the mouse in vivo samples and RAW 264.7 cells, respectively. Gene expression levels were quantified using the 2^−ΔΔCt^ method. The primer sequences used are listed in Table 1.

**TABLE 1:** Sequence of the primers utilized in this work.

	Primers used for quantifying the expression of mRNAs			
q‐PCR	Gene name	Species	Forward primer (5′ → 3′)	Reverse primer (5′ → 3′)
	*Asxl1*	Mouse	GGGCACCCAAGGAATATTTGC	TGGTGTCATTGGAGCATCTGA
	*Kim1*		AAACCAGAGATTCCCACACG	GTCGTGGGTCTTCCTGTAGC
	*Il6*		ACAAAGCCAGAGTCCTTCAGAGAG	TTGGATGGTCTTGGTCCTTAGCCA
	*Il1b*		CCTTCCAGGATGAGGACATGA	AACGTCACACACCAGCAGGTT
	*Edn1*		GGCCCAAAGTACCATGCAGA	TGCTATTGCTGATGGCCTCC
	*Ccl7*		AAGATCCCCAAGAGGAATCTCAAG	CAGACTTCCATGCCCTTCTTTG
	*Csf3*		ATGGCTCAACTTTCTGCCCAG	CTGACAGTGACCAGGGGAAC
	*Lcn2*		TCTCTGTCCCCACCGACCAA	ACAGGTGGATGGGGAGTGCT
	*Nos2*		CACCTTGGAGTTCACCCAGT	ACCACTCGTACTTGGGATGC
	*β‐Actin*		AAGATCAAGATCATTGCTCCTCCTG	AAACGCAGCTCAGTAACAGTCC
ChIP	Primers used to analyze the immunopricicpitated chromatin			
	Gene name	Species	Forward primer (5′ → 3′)	Reverse primer (5′ → 3′)
	*Il6* promoter (H3K27me3 and H3K4me3)	Mouse	ATGCTCAAGTGCTGAGTCAC	GATTGCACAATGTGACGTCG
	*Il1b* promoter (H3K27me3)		TACACTGATACCTGCATACTGTGTGTG	GGGTTTTCCTGGGTTAGCTGTAC
	*Il1b* promoter (H3K4me3)		AGGGACTCCTACAGAATGCAATGGT	TGCTCTGGTTGCTCTCTGTTGACT
	*Csf3* promoter (H3K27me3 and H3K4me3)		TACCCATGATTGGGCAAGGC	GAGTCGATGCATGCTGAGGT
	*Lcn2* promoter (H3K27me3 and H3K4me3)		AGGACTCCTACAGGGTTATG	TGTGCAAGGTTGAGCAACAG
	*Edn1* promoter (H3K27me3 and H3K4me3)		GGACAGTTCTCCTTTGCTTTCC	CACACCCCTTTCTGACCTCC
	*Ccl7* promoter (H3K27me3 and H3K4me3)		GATTCCAAGATCTGAATCCTTCTG	ACATGATGCCTTAAAGTGTCCAG
	*Nos2* promoter (H3K27me3 and H3K4me3)		GAGTCTCAGTCTTCAACTCCCTGT	TGGGAACAGTTATGCAAAATAGC

Abbreviation: ChIP, chromatin immunoprecipitation.

### 2.7 ELISA

The concentration of IL‐6 was measured using a Mouse IL‐6 Uncoated ELISA Kit with plates (88‐7064‐22; Thermo Fisher Scientific, Waltham, MA) according to the instructions of the manufacturer. All samples were measured in technical duplicates across three independent experiments.

### 2.8 RNA sequencing sample preparation, library construction, and sequencing

Total RNA was isolated as described above from four groups of RAW 264.7 cells: si‐negative control (si‐NC) vehicle, si‐*Asxl1* vehicle, si‐NC LPS, and si‐*Asxl1* LPS. RNA integrity was assessed using the Agilent RNA 6000 Nano kit (5067‐1511; Agilent) on a Bioanalyzer (Agilent). All samples showed RNA integrity numbers between 9.9 and 10.0, confirming their suitability for mRNA sequencing. For each sample, 2000 ng of total RNA was used to prepare sequencing libraries with the NEBNext Ultra II RNA Library Prep Kit for Illumina and NEBNext Poly(A) mRNA Magnetic Isolation Module (7770L and E7490; New England Biolabs, Ipswich, MA), following the instructions of the manufacturer. The final PCR amplification was performed using 12 thermal cycles. Library concentration and fragment size distribution were evaluated with the Agilent DNA 7500 Kit (5067‐1506; Agilent, Santa Clara, CA) on a Bioanalyzer. All libraries met quality requirements for next‐generation sequencing.

The libraries were pooled, denatured, neutralized, and subjected to next‐generation sequencing on the NextSeq 500 System (20024906; Illumina, San Diego, CA) using paired‐end reads of 36 bases. FASTQ files were generated and quality‐checked using CLC Genomics Workbench 23.02 (QIAGEN). Over 99% of reads exhibited a PHRED quality score >20, confirming successful data acquisition. The total number of paired‐end reads per sample ranged from approximately 31–41 million.

### 2.9 Bioinformatics analysis

Bioinformatics analysis was conducted using CLC Genomics Workbench software version 23.02 (QIAGEN). The workflow included statistical analysis, quantification of gene expression, and generation of heatmaps and principal component analysis plots, followed by mapping to the GCRm39 reference genome using general transfer format files. Additional data processing and extraction of genes of interest were performed in Microsoft Excel 2019 (Redmond, WA). Cluster dendrograms were created based on the expression (counts per million) of all genes using the R programming language (version 4.1.1). Distance thresholds of 5000 or 4000 were applied for clustering. The web tool Venny version 2.1.0 (available at https://bioinfogp.cnb.csic.es/tools/venny/) was used to identify genes that varied according to LPS stimulation and ASXL1 status, using the following selection criteria: maximum group mean of 1 and false discovery rate (FDR) *p*‐value <0.01. Gene ontology enrichment and transcriptional regulatory relationships unraveled by sentence‐based text mining (TRRUST) analyses were performed using Metascape (Zhou et al., 2019), applying the gene lists filtered according to these thresholds.

### 2.10 Phagocytosis assay

RAW 264.7 cells (1.0 × 10^5^ cells/well) were cultured in six‐well plates. *Asxl1* expression was knocked down using siRNA transfection, and cells were subsequently stimulated with LPS (100 ng/mL). Latex beads coated with fluorescein isothiocyanate (FITC) –rabbit IgG (500290; Cayman Chemical, Ann Arbor, MI) were added at a 1:2000 dilution and incubated for 3 h, followed by two washes with phosphate‐buffered saline. The proportion of beads phagocytosed by RAW 264.7 cells was quantified using flow cytometry (CytoFLEX; Beckman Coulter, Brea, CA). For imaging, cells were fixed with 4% paraformaldehyde for 20 min, and images were acquired at 200× magnification using a KEYENCE BZ‐X710 microscopy system.

### 2.11 Co‐culture and cytotoxicity assay

Cytotoxicity was evaluated using the Annexin V‐APC/PI Apoptosis Assay—which was used to assess cell apoptosis—and the ViaLight Plus™ BioAssay. MCT cells (1.0 × 10^4^ cells/well) were cultured in six‐well plates. After 24 h, the medium was replaced with conditioned medium collected from four groups of RAW 264.7 cells: si‐NC vehicle, si‐*Asxl1* vehicle, si‐NC LPS, and si‐*Asxl1* LPS. Cells were washed twice with cold Cell Staining Buffer (420201; BioLegend, San Diego, CA) and resuspended in Annexin V Binding Buffer (422201; BioLegend, San Diego, CA). Annexin V‐allophycocyanin (5 μL; 640920; BioLegend, San Diego, CA) and propidium iodide (10 μL) staining solution (13‐6990; Tonbo Biosciences, Bethesda, MD) were then added, and the samples were incubated for 15 min at room temperature (25°C) in the dark. The percentage of apoptotic cells was quantified by flow cytometry (CytoFLEX; Beckman Coulter, Brea, CA).

Cell viability was assessed using the ViaLight Plus™ Cell Proliferation and Cytotoxicity BioAssay Kit (LT07‐121; LONZA, Basel, Switzerland). MCT cells (1.0 × 10^3^ cells/well) were cultured in 96‐well plates (167008; Thermo Fisher Scientific, Waltham, MA). The medium was then replaced with conditioned medium obtained from the four groups of RAW 264.7 cells described above. ViaLight Plus reagents were added to each well according to the instructions of the manufacturer, and luminescence was measured using a microplate reader to determine cell viability.

### 2.12 Chromatin immunoprecipitation (ChIP)

Four groups of RAW 264.7 cells—si‐NC vehicle, si‐*Asxl1* vehicle, si‐NC LPS, and si‐*Asxl1* LPS—were used for chromatin immunoprecipitation. Cells (1.0 × 10^5^ per sample) were fixed with 1% formaldehyde for 10 min at room temperature, and the reaction was quenched with glycine (final concentration: 125 mM). Dynabeads Protein G (10003D; Thermo Fisher Scientific, Waltham, MA) and Dynabeads M‐280 Sheep Anti‐Mouse IgG (11201D; Thermo Fisher Scientific, Waltham, MA) were preabsorbed with 0.5% bovine serum albumin, with or without the indicated antibodies (detailed below), for 1 h to overnight at 4°C with rotation for H3K27me3 and H3K4me3 immunoprecipitation, respectively. After washing with phosphate‐buffered saline, cells were suspended in swelling buffer (20 mM HEPES, 1.5 mM MgCl_2_, 10 mM KCl, 0.1% NP‐40, and 1 mM dithiothreitol) for 20 min on ice, then resuspended in sonication buffer (1× Shearing Buffer; Covaris No. 520154). Lysates were sonicated using a HANDY Branson SFX150 sonicator for 45 cycles (amplitude: 30%; ON/OFF = 2 s/3 s, pulse mode) to obtain DNA fragments. The lysates were centrifuged to remove insoluble debris, and the supernatants were collected. Abcam radioimmunoprecipitation assay buffer (50 mM Tris–HCl, 150 mM NaCl, 2 mM ethylenediaminetetraacetic acid [EDTA], 1% NP‐40, and 0.5% sodium deoxycholate, 0.1% sodium dodecyl sulfate [SDS]) was then added. Samples were precleared with preabsorbed Dynabeads (without antibody) for 1 h at 4°C with rotation. After removing the beads, supernatants were collected, and 9% of each sample was separated as chromatin input for normalization. The remaining supernatant was incubated overnight at 4°C with preabsorbed Dynabeads containing antibodies for immunoprecipitation. Samples were washed three times with low‐salt buffer (20 mM Tris–HCl, 150 mM NaCl, 2 mM EDTA, 1% Triton X‐100, and 0.1% SDS) and once with high‐salt buffer (20 mM Tris–HCl, 500 mM NaCl, 2 mM EDTA, 1% Triton X‐100, and 0.1% SDS). Elution buffer (10 mM Tris–HCl, 300 mM NaCl, 5 mM EDTA, and 0.5% SDS) was added, and samples were incubated for 15 min at 65°C to detach antibodies from the beads. Supernatants were collected, and both input and immunoprecipitation samples were treated with proteinase K (3115887001; Merck, Darmstadt, Germany) for 6 h at 37°C, followed by 12 h at 65°C to reverse cross‐linking. DNA was purified by ethanol precipitation and analyzed by quantitative real‐time PCR. Immunoprecipitated DNA was normalized to its respective chromatin input. The following antibodies and control IgGs were used: H3K27me3 (C36B11; Cell Signaling Technology, Danvers, MA), H3K4me3 (GTX50897; GeneTex, Irvine, CA), normal rabbit IgG (011‐000‐003; Jackson ImmunoResearch Labs, West Grove, PA), and normal mouse IgG (sc‐2025; Santa Cruz Biotechnology, Dallas, TX). Primer sequences are listed in Table 1.

### 2.13 Statistical analysis

All data are presented as mean ± standard deviation. Statistical analyses were performed using Prism version 10 (GraphPad Software, San Diego, CA). Comparisons among four groups were conducted using one‐way analysis of variance followed by Dunnett's multiple comparisons post hoc test. Comparisons between two groups were made using the unpaired *t*‐test. A *p* < 0.05 was considered statistically significant.

## 3 RESULTS

### 3.1 ASXL1‐positive M1 macrophages increased significantly in the interstitium of male mouse uIRI kidneys in the acute phase

At 24 h after reperfusion, *Kim1* mRNA levels were significantly elevated 196.6 ± 130.1‐fold (*p* = 0.014) in uIRI kidneys compared with sham‐operated mice (Figure 1*a*). KIM1 serves as a reliable biomarker of acute renal tubular injury (Han et al., 2002). In contrast, *Asxl1* mRNA levels did not change between uIRI and sham kidneys (*p* = 0.394) (Figure 1a). Hematoxylin and eosin staining also demonstrated a greater infiltration of inflammatory cells in the interstitium of uIRI kidneys than in sham kidneys. Periodic acid–Schiff staining revealed acute pathological changes, including brush border loss, cast formation, and tubular dilatation. Immunohistochemical staining for CD68, a macrophage marker, showed substantial macrophage accumulation in the interstitium of uIRI kidneys (Figure 1b).

For more analysis focusing on ASXL1 expression of macrophages, we conducted the flow cytometry analysis. It revealed that M1 macrophages (CD11b^+^‐F4/80^+^‐CD86^+^ cells) was significantly increased in uIRI kidneys (*p* = 0.002) (Figure 1c). The ratio of ASXL1 positive macrophages of total macrophages was also significantly higher in uIRI kidneys compared in sham kidneys (10.1 ± 4.1% vs. 2.1 ± 1.8%, *p* = 0.005) (Figure 1d,e). Besides, the number of ASXL1 positive macrophages per kidney weight was significantly elevated 27.6 ± 14.8‐fold (*p* = 0.007) in uIRI kidneys compared to sham kidneys (Figure 1f). In addition, in uIRI kidneys, the ratio of ASXL1 positive M1 macrophages among total M1 macrophages was significantly higher compared to that of ASXL1 positive non‐M1 macrophages among total non‐M1 macrophages (23.4 ± 9.7% vs. 7.3 ± 3.6%, *p* = 0.001) (Figure 1g,h).

Immunofluorescence analysis for ASXL1 and CD68 also revealed that ASXL1‐positive and negative macrophages were mixed in uIRI kidneys, and ASXL1‐positive macrophages were more abundant in the interstitium of uIRI kidneys compared with sham kidneys (*p* < 0.001) (Figure 1h,i). In addition, ASXL1 was stained in both the nucleus and cytoplasm in macrophages (Figure 1i). ASXL1 immunofluorescence was confirmed in the germinal epithelial cells in the testis, a known positive control for ASXL1 (Fagerberg et al., 2014) (Figure S2A). On the other hand, ASXL1 was also stained in tubular epithelial cells and interstitial cells other than macrophages. However, double immunofluorescence staining for ASXL1 and KIM1 demonstrated no clear difference in ASXL1 staining pattern between normal and damaged tubular epithelial cells (Figure S2B). In addition, double immunofluorescence staining for ASXL1 and PDGFRβ also showed no clear difference in ASXL1 staining pattern in PDGFRβ positive cells between normal and uIRI kidneys. Nearly all PDGFRβ positive cells other than glomeruli were ASXL1 positive in both normal and uIRI kidneys (Figure S2C).

**FIGURE 1:**
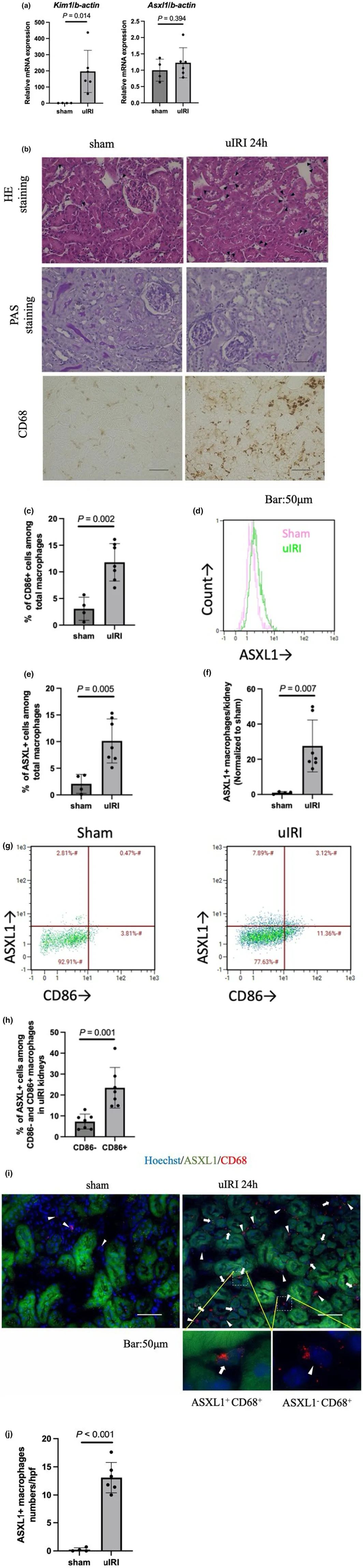
ASXL1 expression in the mouse uIRI model in the acute phase (24 h). (a) RNA isolated from sham and uIRI kidneys was analyzed by quantitative real‐time PCR for *Kim1* and *Asxl1* mRNA levels, normalized to β‐actin. (b) Representative HE, PAS staining, and immunohistochemistry for CD68, a macrophage marker, in sham and uIRI kidneys. Scale bar = 50 μm. Original magnification: 400×. (c–h) Flow cytometry of kidney single‐cell suspensions showing (c) the ratio of M1‐type macrophages (CD11b^+^‐F4/80^+^‐CD86^+^), (d) the representative image of histogram of ASXL1 expression in total macrophages (CD11b^+^‐F4/80^+^), (e) the ratio of ASXL1 positive macrophages (CD11b^+^‐F4/80^+^‐ASXL1^+^) of total macrophages, (f) relative counts for ASXL1 positive macrophages per kidney weight, (g) representative dot plots of CD86 and ASXL1 in total macrophages (CD11b^+^‐F4/80^+^), (h) ASXL1 positive ratio of macrophages was compared between CD86^−^ (non‐M1‐type) and CD86^+^ (M1‐type) macrophages in uIRI kidneys (*n* = 6). (*I*) Representative immunofluorescence images showing ASXL1 (green) and CD68 (red) in sham and uIRI kidneys. While ASXL1‐positive macrophages (white arrows) were scarcely detected in sham kidneys, they were increased in uIRI kidneys. On the other hand, ASXL1‐negative macrophages (white arrowheads) were present in both sham and uIRI kidneys. Nuclei were stained with Hoechst (blue). Scale bar = 50 μm. Original magnification: 400×. ASXL1‐positive and ‐negative macrophages in the uIRI kidney imaged at 1000× magnification. (j) Quantification showing a significant increase in ASXL1‐positive macrophages in the interstitium of uIRI kidneys compared with sham kidneys. Sham group, *n* = 4; uIRI group, *n* = 6. Data are presented as mean ± standard deviation. Unpaired two‐tailed *t*‐test (a, c, e, f, h, and j). HE, hematoxylin and eosin; hpf, high‐power field; mRNA, messenger RNA; PAS, periodic acid–Schiff; PCR, polymerase chain reaction; uIRI, unilateral ischemia–reperfusion injury.

### 3.2 *Asxl1* regulates IL‐6 expression in LPS‐stimulated RAW 264.7 macrophages

We silenced *Asxl1* expression in RAW 264.7 cells and stimulated them with LPS (100 ng/mL) for 24 h. LPS stimulation reduced Asxl1 expression by 38.2 ± 11.0% (*p* = 0.002). Knockdown efficiency of *Asxl1* at the mRNA level was 41.9 ± 4.1% (*p* < 0.001) in the vehicle group and 45.2 ± 2.0% (*p* = 0.014) in the LPS‐stimulated group (Figure 2a). LPS stimulation markedly induced *Il6* expression (*p* = 0.007), a characteristic M1 macrophage marker, and *Asxl1* silencing further enhanced *Il6* mRNA expression by 5.0 ± 0.46‐fold compared with si‐NC M1 macrophages (*p* < 0.001) (Figure 2b). The concentration of IL‐6 in the culture supernatant of *Asxl1*‐knockdown M1 macrophages was also higher than that in si‐NC M1 macrophages (2.5 ± 0.01‐fold, *p* < 0.001) (Figure 2c).

**FIGURE 2:**
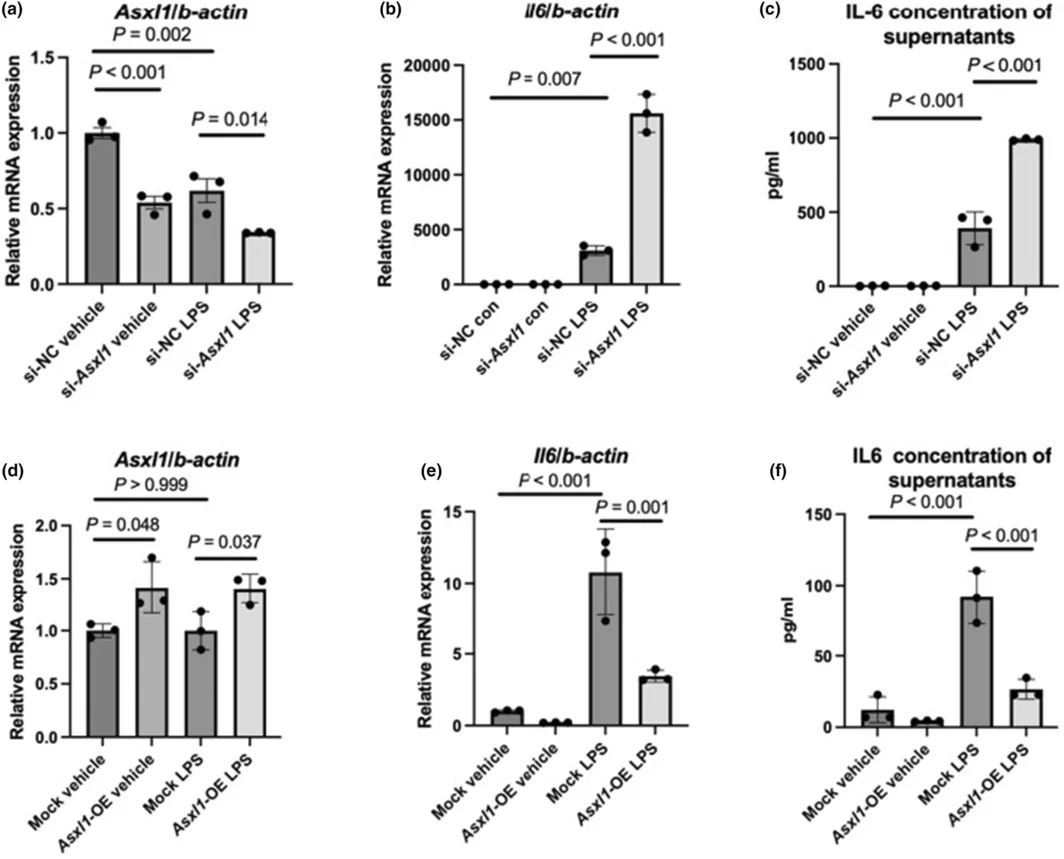
Knocking down *Asxl1* enhances IL‐6 expression and overexpression of *Asxl1* suppresses IL‐6 expression in LPS‐stimulated macrophages. (a, b) Quantitative real‐time PCR analysis showing *Asxl1* (a) and *Il6* (b) mRNA expression in RAW 264.7 cells under *Asxl1* knockdown with vehicle and LPS stimulation conditions. mRNA levels were normalized to β‐actin. (c) ELISA analysis showing IL‐6 concentrations in culture supernatants. *Asxl1* knockdown enhanced IL‐6 expression in LPS‐stimulated RAW 264.7 cells at the protein level. (d, e) Quantitative real‐time PCR analysis showing *Asxl1* (d) and Il6 (e) mRNA expression in RAW 264.7 cells under *Asxl1* overexpression with vehicle and LPS stimulation conditions. mRNA levels were normalized to β‐actin. (f) ELISA analysis showing IL‐6 concentrations in culture supernatants. *Asxl1* overexpression inhibited IL‐6 expression in LPS‐stimulated RAW 264.7 cells at the protein level. Comparisons among four groups were performed using one‐way analysis of variance followed by Dunnett's multiple comparisons post hoc test. The above data represent three independent repeated experiments, and the data are presented as mean ± standard deviation; *n* = 3. ELISA, enzyme‐linked immunosorbent assay; IL‐6, interleukin‐6; LPS, lipopolysaccharide; mRNA, messenger RNA; NC, negative control; OE, overexpression; PCR, polymerase chain reaction.

In addition, we overexpressed *Asxl1* expression in RAW 264.7 cells and stimulated them with LPS (100 ng/mL) for 24 h. Transfection with *Asxl1* plasmid increased its mRNA levels by 1.41 ± 0.20‐fold (*p* = 0.048) in the vehicle group and 1.40 ± 0.14‐fold (*p* = 0.038) in the LPS‐stimulated group (Figure 2d). LPS stimulation did not change Asxl1 expression (*p* > 0.999) (Figure 2d). *Asxl1* overexpression suppressed *Il6* expression by 0.32 ± 0.03‐fold compared with mock LPS‐induced M1 macrophages (*p* = 0.001) (Figure 2e). The concentration of IL‐6 in the culture supernatant of *Asxl1*‐OE M1 macrophages was also lower than that in mock LPS‐induced M1 macrophages (0.29 ± 0.06‐fold, *p* < 0.001) (Figure 2f).

Thus, these findings indicated that ASXL1 functions as a negative regulator of IL‐6 production in M1 macrophages.

### 3.3 ASXL1 inhibits the inflammatory response in LPS‐induced M1 macrophages

To comprehensively elucidate the transcriptional mechanisms underlying macrophage activation, RNA sequencing was performed on si‐NC and si‐*Asxl1* RAW 264.7 cells treated with either vehicle or LPS (100 ng/mL) for 24 h. Principal component analysis demonstrated clear clustering of the groups (Figure 3a). RNA sequencing analysis identified 2667 genes upregulated following LPS stimulation (si‐NC + vehicle vs. si‐NC + LPS) and 637 genes upregulated upon *Asxl1* knockdown (si‐NC + LPS vs. si‐*Asxl1* + LPS), based on the criteria of FDR *p* < 0.01, maximum group mean >1, and fold change >1.0. Among these, 229 genes were shared between the two groups (Figure 3b) and were considered to be negatively regulated by ASXL1 in M1 macrophages. These genes are shown in the heatmap (Figure S3). The top 10 significant differentially expressed genes (DEGs) were *Il6*, *Ccl7*, *Lancl3*, *A530064D06Rik*, *Sema3e*, *Edn1*, *Il1b*, *Zfp811*, *Klra2*, and *Ifi209* (in descending order). Gene ontology enrichment analysis revealed that inflammatory pathways—such as response to virus, innate immune response, response to molecules of bacterial origin, defense response to protozoan, influenza A response, positive regulation of DNA‐binding transcription factor activity, and inflammatory response—were upregulated following *Asxl1* silencing in M1 macrophages (Figure 3c). The representative genes for each ontology are listed in Table 2. Within these pathways, inflammatory genes including *Il1b*, *Edn1*, and *Ccl7* were validated by quantitative PCR (Figure 3d). Silencing *Asxl1* enhanced the mRNA expression of *Il1b*, *Edn1*, and *Ccl7* by 3.6 ± 0.25‐fold (*p* < 0.001), 4.6 ± 0.41‐fold (*p* < 0.001), and 1.6 ± 0.07‐fold (*p* < 0.001), respectively, compared with si‐NC M1 macrophages (Figure 3e). Furthermore, TRRUST analysis suggested that transcription factors such as nuclear factor kappa‐light‐chain‐enhancer of activated B cells (NF‐κB), CCAAT/enhancer‐binding protein beta, and signal transducer and activator of transcription 1 act as upstream regulators of these inflammatory response pathways (Figure 3e).

**FIGURE 3:**
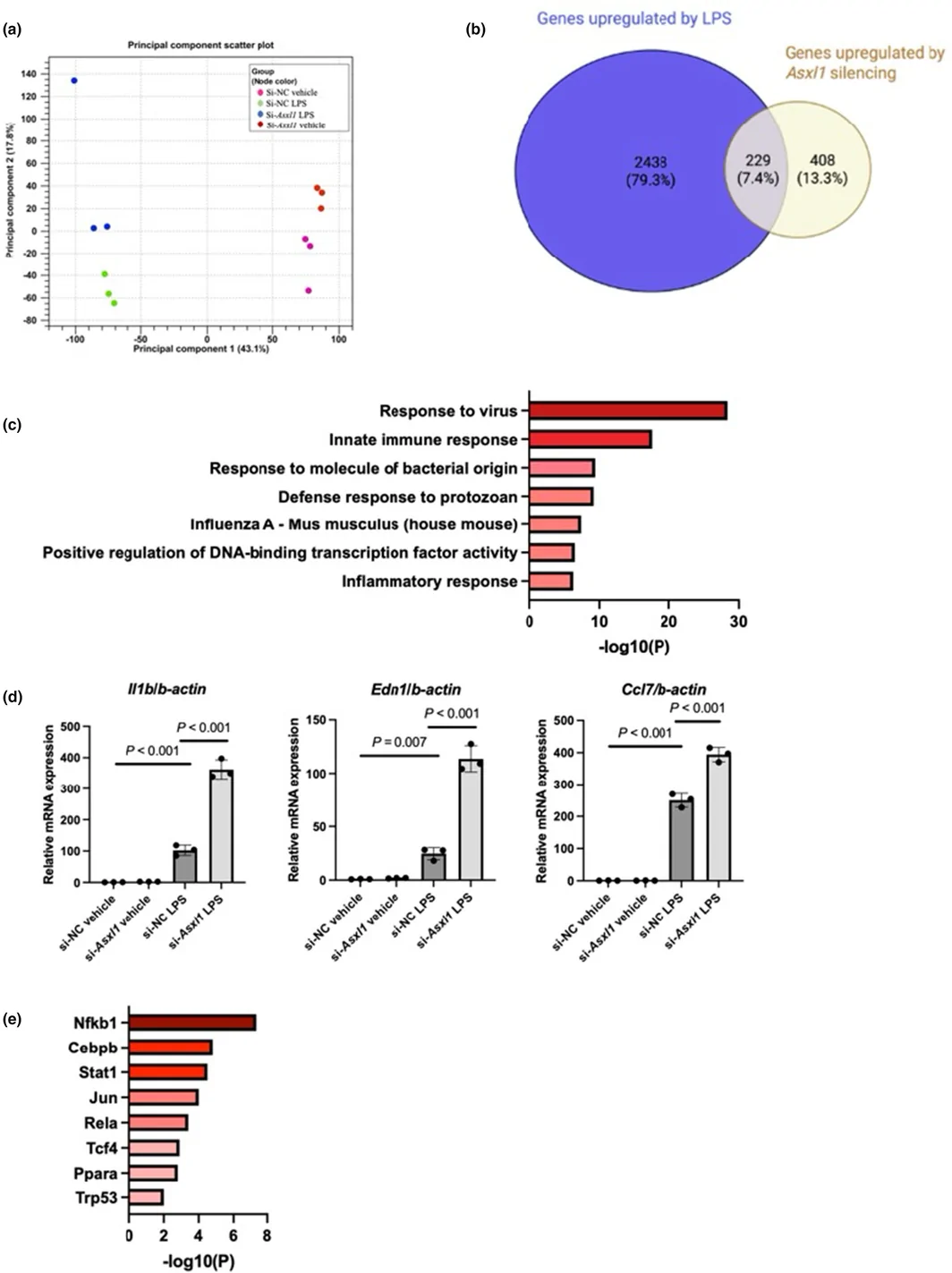
Transcriptome analysis of RAW 264.7 cells identifying genes upregulated by both LPS stimulation and *Asxl1* silencing. RNA sequencing was performed on four groups of RAW 264.7 cells: si‐NC vehicle, si‐*Asxl1* vehicle, si‐NC LPS, and si‐*Asxl1* LPS (*n* = 3 per group). (a) Principal component analysis showing distinct gene expression patterns among groups: si‐NC vehicle (pink), si‐*Asxl1* vehicle (red), si‐NC LPS (green), and si‐*Asxl1* LPS (blue). (b) Venn diagram showing genes upregulated by both LPS stimulation and *Asxl1* knockdown (false discovery rate *p* < 0.01; maximum group mean >1; fold change >1.0). A total of 229 genes overlapped between the two groups. (c) Gene ontology pathway analysis of these genes. (d) Quantitative real‐time PCR validation of *Il1b*, *Edn1*, and *Ccl7* mRNA expression in RAW 264.7 cells, normalized to β‐actin. Comparisons among four groups were made using one‐way analysis of variance followed by Dunnett's multiple comparisons post hoc test. Data are presented as mean ± standard deviation; *n* = 3. (e) TRRUST analysis of these genes showing associated transcriptional regulators. *n* = 3 per group. LPS; lipopolysaccharide; NC, negative control; mRNA; messenger RNA; PCR, polymerase chain reaction; TRRUST, transcriptional regulatory relationships unraveled by sentence‐based text mining.

**TABLE 2:** Molecular functions that were enriched for genes whose expression was elevated by both LPS stimulation and Asxl1 silencing.

Description	Gene symbols	−log *p* value
Response to virus	*Bcl3, Casp1, Ifi44l, Hsp90aa1, Cxcl10, Ifi203, Ifi204, Ifit1, Ifit2, Ifit3, **Il6*** ^*^, *Lgals9, Mx1, Mx2, Pou2f2, Eif2ak2, Trim30a, Stat2, Cd40, Oasl2, Irgm2, Samhd1, Isg20, Rsad2, Nmi, Ifih1, Parp9, Tlr3, Trim25, Treml4, Gbp7, Ddx60, **Ifi209*** ^*^, *Slfn9, Slfn8, Trim12c, Ifi211, Ifi208, Mndal*	28.42
Innate immune response	*Casp1, Cfb, Ifi204, Ifit1, Ifit2, Ifit3, Cd47, Cd180, Mx1, Mx2, Cfp, Eif2ak2, Rel, Trim30a, Ccl12, Ccl22, **Ccl7*** ^*^ *, Stat2, Tlr1, Oasl2, Irgm2, Gbp3, Samhd1, Isg20, Rsad2, Iigp1, Nmi, Cxcl16, Herc6, Tasl, Ifih1, Parp9, Tlr3, Trim25, Treml4, Gbp7, Gbp9, Fcgr4, Nlrc4, Trim12c, Parp14*	17.5
Response to molecule of bacterial origin	*Adam9, Adm, Casp1, Cd36, Cxcl10, **Il1b*** ^*^ *, **Il6*** ^*^ *, Lgals9, Cd180, Serpine1, Eif2ak2, Ptgs2, Tlr1, Cmpk2, Zfp36, Irgm2, Gbp3, Stap1, Cxcl16, Nfkbiz, Cyrib, Fcgr4*	9.47
Defense response to protozoan	*Bcl3, **Il6*** ^*^ *, Cd40, Irgm2, Gbp3, Iigp1, Gbp7, Gbp9, Tgtp2*	9.25
Influenza A	*Casp1, Cxcl10, **Il1b*** ^*^ *, **Il6*** ^*^ *, Mx1, Mx2, Eif2ak2, Ccl12, Stat2, Rsad2, Ifih1, Tlr3, Trim25*	7.4
Positive regulation of DNA‐binding transcription factor activity	*Cd36, **Edn1*** ^*^ *, Ifrd1* ^*^ *, **Il1b*** ^*^ *, **Il6*** ^*^ *, Eif2ak2, Trim30a, Cd40, Tfrc, Tlr3, Trim25, Camk1d, Nlrc4, Trim12c*	6.53
Inflammatory response	*C3ar1, Casp1, Cxcl10, **Il1b*** ^*^ *, **Il6*** ^*^ *, Cd47, Lipa, Cd180, Rel, Ccl12, Ccl22, **Ccl7*** ^*^ *, Serpinb9, Tlr1, Tmed2, Nampt, Nfkbiz, Tlr3, Camk1d, Fcgr4, Nlrc4*	6.36

*Note*: *Adam9*, ADAM metallopeptidase domain 9. *Adm*, adrenomedullin. *Bcl3*, B cell. leukemia/lymphoma 3. *C3ar1*, complement component 3a receptor 1. *Camk1d*, calcium/calmodulin‐dependent protein kinase ID. *Casp1*, caspase 1. *Ccl12*, C‐C motif chemokine ligand 12. *Ccl7*, C‐C motif chemokine ligand 7. *Cd180*, CD180 antigen. *Cd36*, CD36 molecule. *Cd40*, CD40 antigen. *Cd47*, CD47 antigen. *Cfb*, complement factor B. *Cmpk2*, cytidine/uridine monophosphate kinase 2. *Cxcl10*, C‐X‐C motif chemokine ligand 10. *Cxcl16*, C‐X‐C motif chemokine ligand 16. *Cyrib*, CYFIP related Rac1 interactor B. *Ddx60*, DExD/H box helicase 60. *Eif2ak2*, eukaryotic translation initiation factor 2‐alpha kinase 2. *Fcgr4*, Fc receptor, IgG, low affinity IV. *Gbp3*, guanylate binding protein 3. *Gbp7*, guanylate binding protein 7. *Gbp9*, guanylate binding protein 9. *Herc6*, hect domain and RLD 6. *Hsp90aa1*, heat shock protein 90, alpha (cytosolic), class A member 1. *Ifi203*, interferon activated gene 203. *Ifi204*, interferon activated gene 204. *Ifi208*, interferon activated gene 208. *Ifi209*, interferon activated gene 209. *Ifi211*, interferon activated gene 211. *Ifi44l*, interferon activated gene 441. *Ifih1*, interferon induced with helicase C domain 1. *Ifit1*, interferon‐induced protein with tetratricopeptide repeats 1. *Ifit2*, interferon‐induced protein with tetratricopeptide repeats 2. *Ifit3*, interferon‐induced protein with tetratricopeptide repeats 3. *Ifrd1*, interferon‐related developmental regulator 1. *Iigp1*, interferon inducible GTPase 1. *Irgm2*, immunity‐related GTPase family M member 2. *Isg20*, interferon‐stimulated protein. *Lgals9*, lectin, galactose binding, soluble 9. *Lipa*, lysosomal acid lipase A. *Mndal*, myeloid nuclear differentiation antigen like. *Mx1*, MX dynamin‐like GTPase 1. *Mx2*, MX dynamin‐like GTPase 2. *Nampt*, nicotinamide phosphoribosyltransferase. *Nfkbiz*, nuclear factor of kappa light polypeptide gene enhancer in B cells inhibitor, zeta. *Nlrc4*, NLR family, CARD domain containing 4. Nmi, N‐myc (and STAT) interactor. *Oasl2*, 2′‐5′ oligoadenylate synthetase‐like 2. Parp14, poly (ADP‐ribose) polymerase family, member 14. *Parp9*, poly (ADP‐ribose) polymerase family, member 9. *Pou2f2*, POU domain, class 2, transcription factor 2. *Ptgs2*, prostaglandin‐endoperoxide synthase 2. *Rel*, reticuloendotheliosis oncogene. *Rsad2*, radical S‐adenosyl methionine domain containing 2. *Samhd1*, SAM domain and HD domain, 1. *Serpinb9*, serine (or cysteine) peptidase inhibitor, clade B, member 9. *Serpine1*, serine (or cysteine) peptidase inhibitor, clade E, member 1. *Slfn8*, schlafen 8. *Slfn9*, schlafen 9. Stap1, signal transducing adaptor family member 1. *Stat2*, signal transducer and activator of transcription 2. *Tasl*, TLR adaptor interacting with endolysosomal SLC15A4. *Tfrc*, transferrin receptor. *Tgtp2*, T cell specific GTPase 2. *Tlr1*, toll‐like receptor 1. *Tlr3*, toll‐like receptor 3. *Tmed2*, transmembrane p24 trafficking protein 2. *Treml4*, triggering receptor expressed on myeloid cells‐like 4. *Trim12c*, tripartite motif‐containing 12C. *Trim25*, tripartite motif‐containing 25. *Trim30a*, tripartite motif‐containing 30A. *Zfp36*, zinc finger protein 36.

^*^ Genes of bold characters are within the top 10 significant differentially expressed genes (DEGs) that were upregulated by LPS stimulation and more upregulated by silencing *Asxl1*.

### 3.4 ASXL1 maintains the anti‐apoptotic effect in LPS‐induced M1 macrophages

RNA sequencing identified 2667 genes upregulated by LPS stimulation (si‐NC + vehicle vs. si‐NC + LPS) and 842 genes downregulated following *Asxl1* knockdown (si‐NC+ LPS vs. si‐*Asxl1* + LPS) with FDR *p* < 0.01, maximum group mean >1, and fold change <1.0. Among these, 283 genes were common to both groups (Figure 4a) and were considered to be positively regulated by ASXL1 in M1 macrophages. These genes are presented in the heatmap (Figure S4). The top 10 significant DEGs were *Lcn2*, *Csf3*, *Pdpn*, *Cstdc4*, *Cxcl2*, *Nos2*, *Pilra*, *Lif*, *Gm10800*, and *Cda* (in descending order). Gene ontology enrichment analysis revealed that pathways related to the IL‐17 signaling pathway, inflammatory response, regulation of inflammatory response, and neutrophil degranulation were downregulated following *Asxl1* knockdown (Figure 4b). Moreover, the Fcγ receptor (FCGR)‐dependent phagocytosis pathway was also suppressed by *Asxl1* knockdown. The representative genes for each ontology are listed in Table 3. Within the IL‐17 signaling pathway, *Lcn2* and *Csf3*—both possessing anti‐apoptotic functions—were among the most significantly downregulated genes. In the inflammatory response pathway, *Nos2* (encoding inducible nitric oxide synthase) was also identified. *Lcn2*, *Csf3*, and *Nos2* expression levels were significantly upregulated by LPS stimulation but markedly downregulated upon *Asxl1* silencing (Figure 4c). Specifically, *Asxl1* knockdown reduced *Lcn2*, *Csf3*, and *Nos2* mRNA levels to 0.71 ± 0.05‐fold (*p* = 0.002), 0.29 ± 0.04‐fold (*p* = 0.002), and 0.76 ± 0.08‐fold (*p* = 0.01), respectively, compared with si‐NC M1 macrophages (Figure 4d). Furthermore, TRRUST analysis identified NF‐κB, FOS, JUN, ETS2, and signal transducer and activator of transcription 3 as upstream transcriptional regulators of these inflammatory and survival‐related pathways (Figure 4d).

**FIGURE 4:**
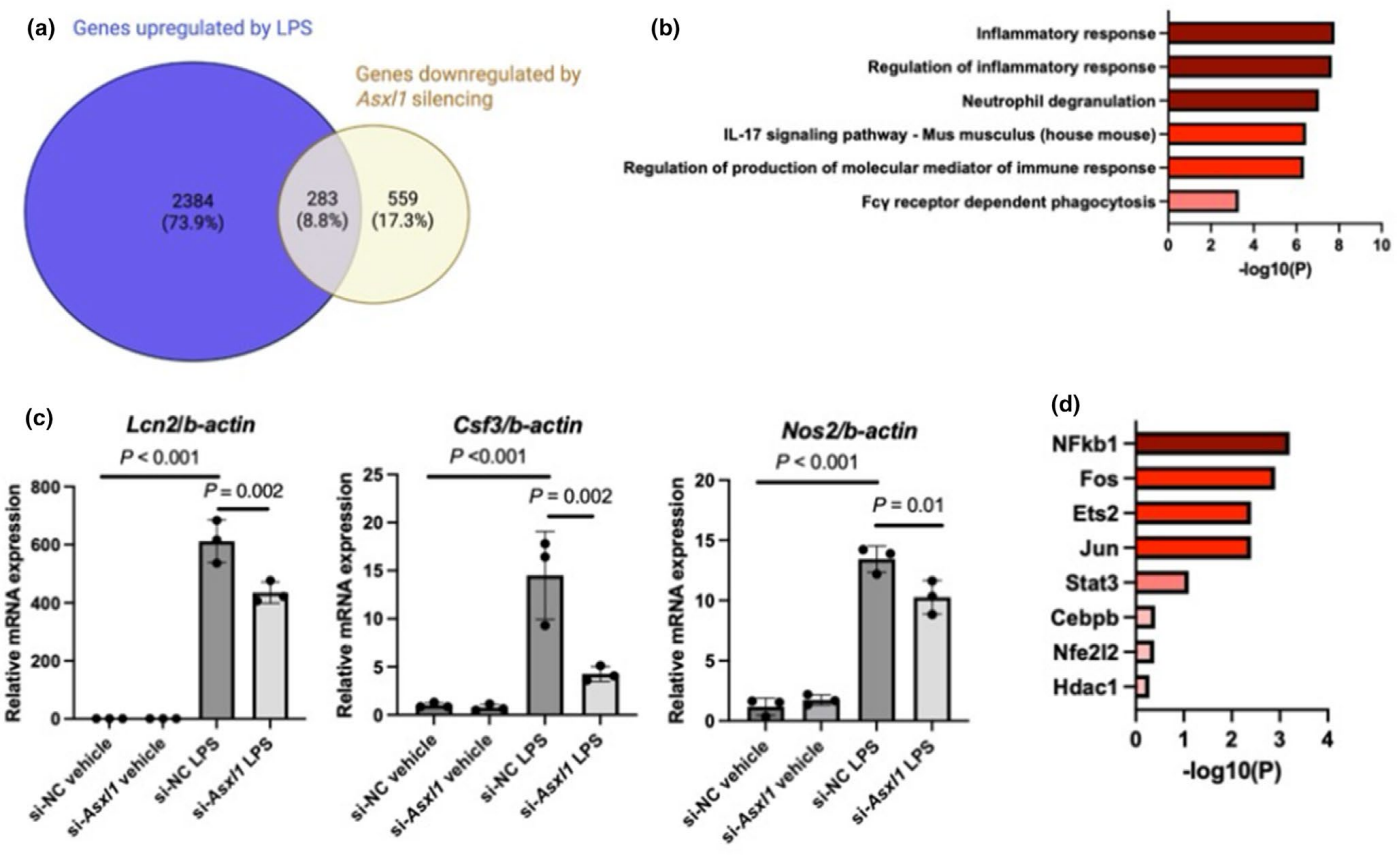
Transcriptome analysis of RAW 264.7 cells identifying genes upregulated by LPS stimulation and suppressed by *Asxl1* silencing. RNA sequencing was performed on four groups of RAW 264.7 cells: si‐NC vehicle, si‐*Asxl1* vehicle, si‐NC LPS, and si‐*Asxl1* LPS. (a) Venn diagram showing genes upregulated by LPS stimulation and downregulated by *Asxl1* knockdown (false discovery rate *p* < 0.01; maximum group mean >1; fold change <1.0). A total of 283 genes overlapped between the two groups. (b) Gene ontology pathway analysis of these genes. (c) Quantitative real‐time PCR validation of *Lcn2*, *Csf3*, and *Nos2* mRNA expression in RAW 264.7 cells, normalized to β‐actin. Comparisons among four groups were made using one‐way analysis of variance followed by Dunnett's multiple comparisons post hoc test. Data are presented as mean ± standard deviation; *n* = 3. (d) TRRUST analysis of these genes showing associated transcriptional regulators. *n* = 3 per group. LPS; lipopolysaccharide; mRNA; messenger RNA; NC, negative control; PCR, polymerase chain reaction; TRRUST, transcriptional regulatory relationships unraveled by sentence‐based text mining.

**TABLE 3:** Molecular functions that were enriched for genes whose expression was elevated in LPS stimulation and suppressed by silencing ASXL1.

Description	Gene symbols	−log *p* value
Inflammatory response	*Adam8, Alox5ap, C5ar1, Cebpb, Ccr1, Fcgr2b, Fosl1, B4galt1, Hk1, Hmox1, Ndst1, Il4ra, Acod1, Jun, **Nos2*** ^*^ *, Pld3, Pstpip1, Ptafr, S100a8, **Cxcl2*** ^*^ *, Tgfb1, Mfhas1, Scyl1, Pld4, Stab1, Tlr13*	7.79
Regulation of inflammatory response	*Adam8, Alox5ap, Cebpb, Socs3, Fcgr2b, Gstp1, H2‐Q2, H2‐Q4, Ier3, Il16, Acod1, Osm, S100a8, Tgfb1, Mfhas1, Ptges, Tradd, Gpsm3, H2‐Q6, Metrnl, Syt11, Nlrx1*	7.66
neutrophil degranulation	*Adam8, C5ar1, Ctsd, Fcgr2b, B4galt1, Gstp1, H2‐Q2, H2‐Q4, **Lcn2*** ^*^ *, Lyz2, Plaur, Ptafr, S100a8, **Cxcl2*** ^*^ *, Slpi, Stk10, Rhof, Dok3, Ormdl3, Arl8a, Mcemp1, Cda, H2‐Q6, Hk3*	7.05
IL‐17 signaling pathway	*Cebpb, **Csf3*** ^*^ *, Fosl1, Jun, **Lcn2*** ^*^ *, S100a8, **Cxcl2*** ^*^ *, Usp25, Ikbke, Tradd*	6.45
Regulation of production of molecular mediator of immune response	*Cd37, Fcgr2b, H2‐Q2, H2‐Q4, Hk1, Hmox1, Il4ra, Jak3, Stx4a, Tgfb1, Litaf, Vsir, H2‐Q6, Arid5a, Nlrx1*	6.35
Fcγ receptor (FCGR) dependent phagocytosis	*Fcgr2b, Myo10, Pld3, Pla2g6, Plpp5, Pld4*	3.3

*Note*: *Acod1*, conitate decarboxylase 1. *Adam8*, ADAM metallopeptidase domain 8. *Alox5*, aparachidonate 5‐lipoxygenase activating protein. *Arid5a*, AT‐rich interaction domain 5A. *Arl8a*, ARF like GTPase 8A. *B4galt1*, beta‐1,4‐galactosyltransferase 1. *C5ar1*, complement C5a receptor 1. *Cd37*, CD37 molecule. *Cda*, cytidine deaminase. *Cebpb*, CCAAT enhancer binding protein beta. *Ctsd*, cathepsin D. *Cxcl2*, C‐X‐C motif chemokine ligand 2. *Dok3*, docking protein 3. *Fcgr2b*, Fc gamma receptor IIb. Fosl1, fos‐like antigen 1. *Gpsm3*, G‐protein signaling modulator 3. *Gstp1*, glutathione S‐transferase, pi 1. *H2‐Q2*, histocompatibility 2, Q region locus 2. *H2‐Q4*, histocompatibility 2, Q region locus 4. *H2‐Q6*, histocompatibility 2, Q region locus 6. *Hk1*, hexokinase 1. *Hk3*, hexokinase 3. *Hmox1*, heme oxygenase 1. *Ier3*, immediate early response 3. *Ikbke*, inhibitor of kappaB kinase epsilon. *Il16*, interleukin 16. *Il4ra*, interleukin 4 receptor, alpha. *Jak3*, Janus kinase 3. *Jun*, jun proto‐oncogene. *Litaf*, LPS‐induced TN factor. *Lyz*, lysozyme. *Mcemp1*, mast cell expressed membrane protein 1. *Metrnl*, meteorin, glial cell differentiation regulator‐like. *Mfhas1*, malignant fibrous histiocytoma amplified sequence 1. Myo10, myosin X. *Ndst1*, N‐deacetylase/N‐sulfotransferase (heparan glucosaminyl) 1. *Nlrx1*, NLR family member X1. *Nos2*, nitric oxide synthase 2, inducible. *Ormdl3*, ORM1‐like 3 (S. cerevisiae). *Osm*, oncostatin M. *Pla2g6*, phospholipase A2, group VI. *Plaur*, plasminogen activator, urokinase receptor. *Pld3*, phospholipase D family member 3. *Pld4*, phospholipase D family member 4. *Plpp5*, phospholipid phosphatase 5. *Pstpip1*, proline‐serine–threonine phosphatase‐interacting protein 1. *Ptafr*, platelet‐activating factor receptor. *Ptges*, prostaglandin E synthase. *Rhof*, ras homolog family member F (in filopodia). *S100a8*, S100 calcium binding protein A8 (calgranulin A). *Scyl1*, SCY1‐like 1 (S. cerevisiae). *Slpi*, secretory leukocyte peptidase inhibitor. *Socs3*, suppressor of cytokine signaling 3. *Stab1*, stabilin 1. *Stk10*, serine/threonine kinase 1. *Stx4a*, syntaxin 4A (placental). *Syt11*, synaptotagmin XI. *Tgfb1*, transforming growth factor, beta 1. *Tlr13*, toll‐like receptor 13. *Tradd*, TNFRSF1A‐associated via death domain. *Usp25*, ubiquitin specific peptidase 25. *Vsir*, V‐set immunoregulatory receptor.

^*^ Genes of bold characters are within the top 10 significant differentially expressed genes (DEGs) that were upregulated by LPS stimulation and suppressed by silencing *Asxl1*.

### 3.5 Knocking down *Asxl1* in M1 macrophages reduces phagocytosis

To further investigate the role of *Asxl1* in macrophage function, we examined the phagocytic capacity of LPS‐stimulated RAW 264.7 cells following *Asxl1* knockdown. Flow cytometry analysis showed that LPS stimulation significantly increased the phagocytosis ratio in M1 macrophages compared with the vehicle group (75.0 ± 4.2% vs. 20.9 ± 5.1%, *p* < 0.001). However, silencing *Asxl1* in M1 macrophages significantly reduced the phagocytosis ratio from 75.0 ± 4.2% to 63.6 ± 0.53% (Figure 5a,b, *p* = 0.01). A representative image of the phagocytosis assay is shown in Figure 5c. Consistent with these findings, gene ontology enrichment analysis of RNA sequencing data from RAW 264.7 cells revealed that the FCGR‐dependent phagocytosis pathway was downregulated by *Asxl1* knockdown (Figure 4c). Together, these results indicate that *Asxl1* contributes to maintaining the phagocytic capacity of M1 macrophages.

**FIGURE 5:**
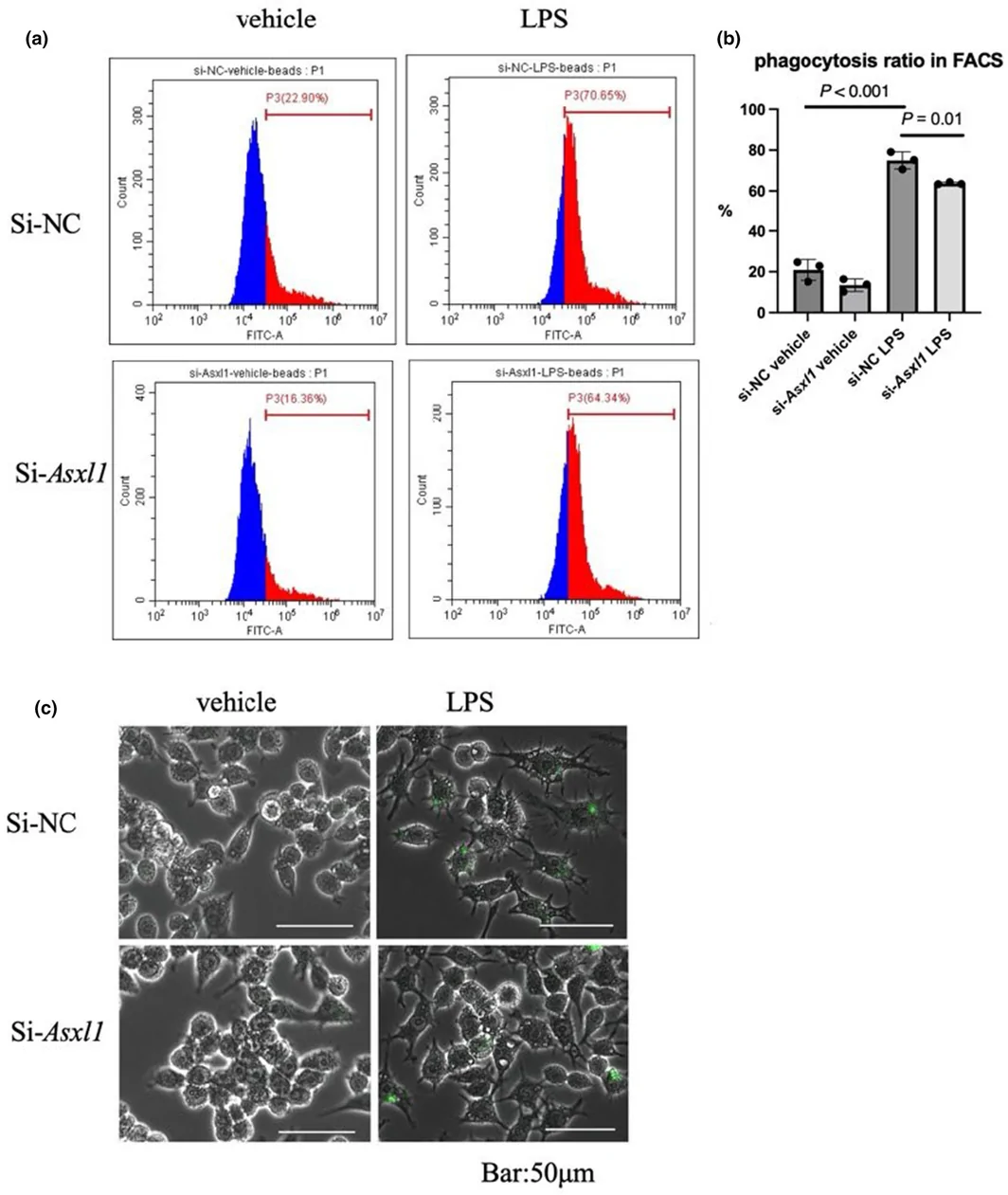
Silencing *Asxl1* in M1 macrophages reduced phagocytic capacity. Phagocytosis assay using latex beads coated with fluorescein isothiocyanate–rabbit IgG was performed in si‐NC and si‐*Asxl1* RAW 264.7 cells treated with vehicle or LPS. (a) Flow cytometry analysis of phagocytosis in each group. (b) Quantification of the phagocytosis ratio determined by flow cytometry. Comparisons among four groups were made using one‐way analysis of variance followed by Dunnett's multiple comparisons post hoc test. The above data represent three independent repeated experiments, and the data are presented as mean ± standard deviation; *n* = 3. (c) Representative fluorescence images showing phagocytosed latex beads in si‐NC and si‐*Asxl1* RAW 264.7 cells treated with vehicle or LPS. FITC‐labeled beads (green) are internalized by RAW 264.7 cells. Scale bar = 50 μm. Original magnification: 200×. IgG, immunoglobulin G; LPS, lipopolysaccharide; NC, negative control.

### 3.6 Silencing *Asxl1* in M1 macrophages induces greater apoptosis and reduces viability of renal tubular epithelial cells in co‐culture

To evaluate the impact of *Asxl1* silencing in M1 macrophages, we analyzed the effects of supernatants from si‐NC or si‐*Asxl1* RAW 264.7 cells treated with vehicle or LPS on apoptosis in mouse renal tubular epithelial (MCT) cells (Figure 6a). Apoptotic cell death was assessed using Annexin V/propidium iodide double staining, where Annexin V‐positive and propidium iodide‐negative cells were defined as apoptotic (Figure 6b). As shown in Figure 6b,c, the percentage of apoptotic MCT cells increased from 7.7 ± 0.5% in the si‐NC vehicle group to 13.8 ± 1.8% in the si‐NC LPS‐treated group (*p* < 0.001). This effect was further exacerbated in the si‐*Asxl1* LPS‐treated group, where apoptosis reached 17.4 ± 0.4% (*p* = 0.005).

**FIGURE 6:**
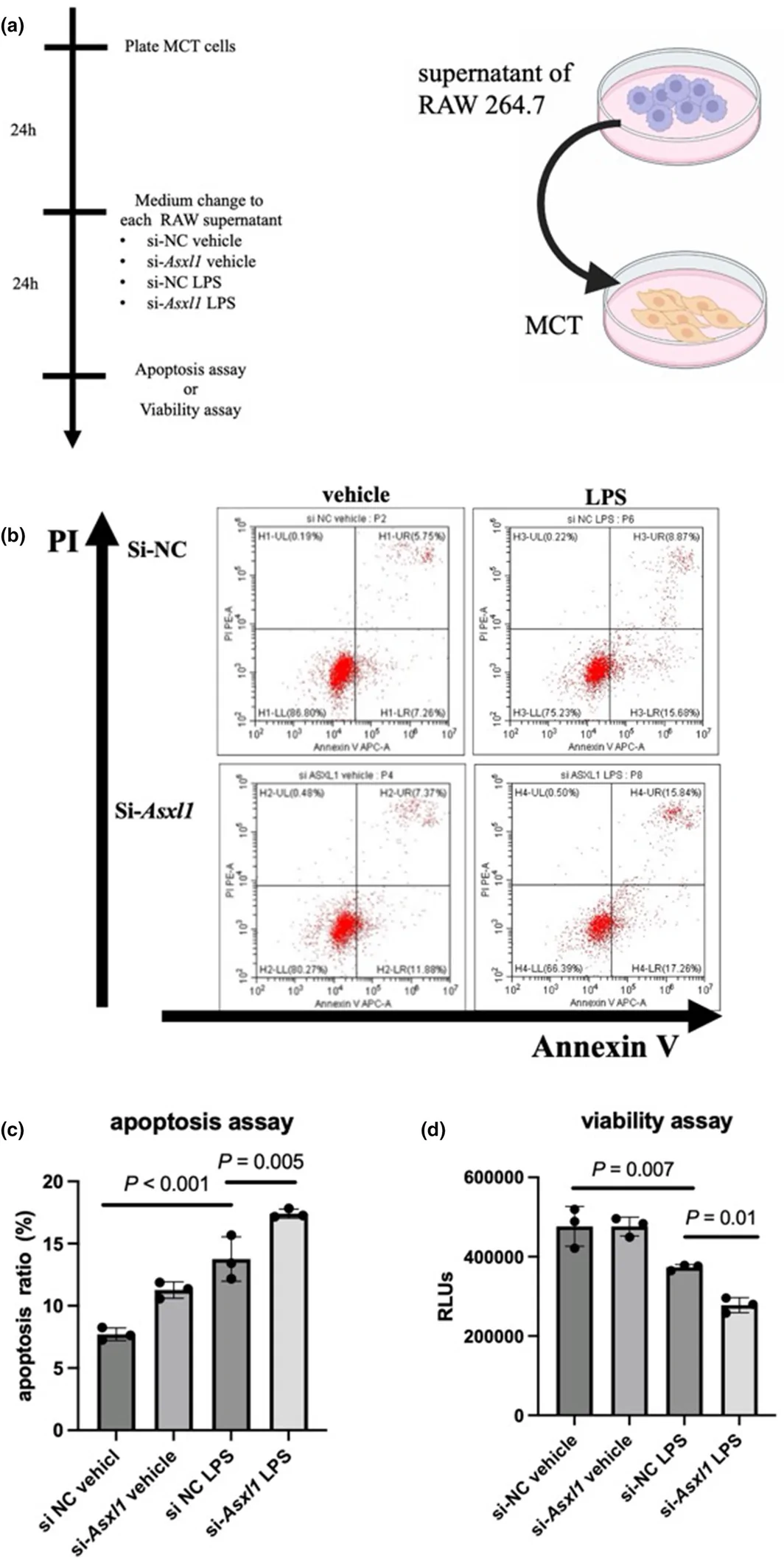
Silencing *Asxl1* in M1 macrophages increases apoptosis and reduces proliferation of renal tubular epithelial cells. (a) Schematic diagram illustrating the workflow of the co‐culture experiment using MCT cells and supernatants from si‐NC or si‐*Asxl1* RAW 264.7 cells treated with vehicle or LPS. (b) Flow cytometry analysis of apoptosis in MCT cells co‐cultured with supernatants from si‐NC or si‐*Asxl1* RAW 264.7 cells treated with vehicle or LPS, assessed by Annexin V/PI staining. (c) Quantification of the percentage of apoptotic MCT cells determined by flow cytometry. (d) Cell viability of MCT cells co‐cultured with supernatants from si‐NC or si‐*Asxl1* macrophages treated with vehicle or LPS, measured using the ViaLight Plus Kit assay. Comparisons among four groups were made using one‐way analysis of variance followed by Dunnett's multiple comparisons post hoc test. The above data represent three independent repeated experiments, and the data are presented as mean ± standard deviation; *n* = 3. LPS, lipopolysaccharide; MCT, mouse renal cortical tubular cells; NC, negative control; PI, propidium iodide; RLUs, relative luminescence units.

We next assessed cell viability using the ViaLight Plus Kit assay (Figure 6d). Co‐culture with supernatants from si‐NC LPS‐treated RAW 264.7 cells reduced MCT cell viability by 21.8 ± 0.05% compared with si‐NC vehicle‐treated cells (*p* = 0.007), whereas supernatants from si‐*Asxl1* LPS‐treated macrophages further decreased cell viability by an additional 25.5 ± 0.05% relative to the si‐NC LPS‐treated group (*p* = 0.01) (Figure 6d).

These results demonstrate that silencing *Asxl1* in M1 macrophages enhances the pro‐apoptotic and cytotoxic effects of macrophage‐derived factors on renal tubular epithelial cells, leading to increased apoptosis and decreased cell viability in co‐culture.

### 3.7 *Asxl1* coordinates H3K27me3 and H3K4me3 levels at promoters of the inflammatory genes and anti‐apoptosis related genes in M1 macrophages

As a chromatin regulator, ASXL1 mediates gene‐specific transcriptional control of inflammatory cytokines in macrophages. To explore the mechanism underlying ASXL1‐mediated regulation, we examined histone modifications (H3K27me3 and H3K4me3) at the promoters of inflammatory genes (*Il6, Il1b*, *Edn1*, *Ccl7*, and *Nos2*) and anti‐apoptosis related genes (*Csf3* and *Lcn2*) *in* si‐NC and si‐*Asxl1* RAW 264.7 cells treated with vehicle or LPS (100 ng/mL) for 24 h. Normal rabbit or mouse IgG was used as a negative control.

We observed that levels of H3K27me3, a repressive histone mark, were reduced at the *Il6* and *Il1b* promoters in LPS‐stimulated cells compared with vehicle‐treated cells to 0.71 ± 0.08‐fold (*p* = 0.04) and 0.56 ± 0.13‐fold (*p* = 0.008), respectively. Knockdown of *Asxl1* further suppressed H3K27me3 levels at these promoters to 0.26 ± 0.23‐fold (*p* = 0.002) and 0.10 ± 0.07‐fold (*p* = 0.004), respectively (Figure 7a,c), which may contribute to enhanced transcription of *Il6* and *Il1b*. We also found that H3K4me3 levels, an active histone mark, were markedly increased at the *Il6* and *Il1b* promoters in LPS‐stimulated macrophages compared with vehicle‐treated cells—1.9 ± 0.54‐fold (*p* = 0.145) and 60.1 ± 10.5‐fold (*p* < 0.001), respectively. On the other hand, silencing *Asxl1* tended to decrease H3K4me3 levels at *Il6* promoters to 0.54 ± 0.32‐fold (*p* = 0.161) in LPS‐stimulated macrophages. However, silencing *Asxl1* did not change H3K4me3 enrichment at *Il1b* promoters (*p* = 0.401) (Figure 7d). These findings suggest that ASXL1 is influencing mainly H3K27me3 levels at the promoters of *Il6* and *Il1b*, and maintains H3K27me3 levels at the promoters of these inflammatory cytokine genes, potentially preventing excessive activation of inflammatory pathways in M1 macrophages.

**FIGURE 7:**
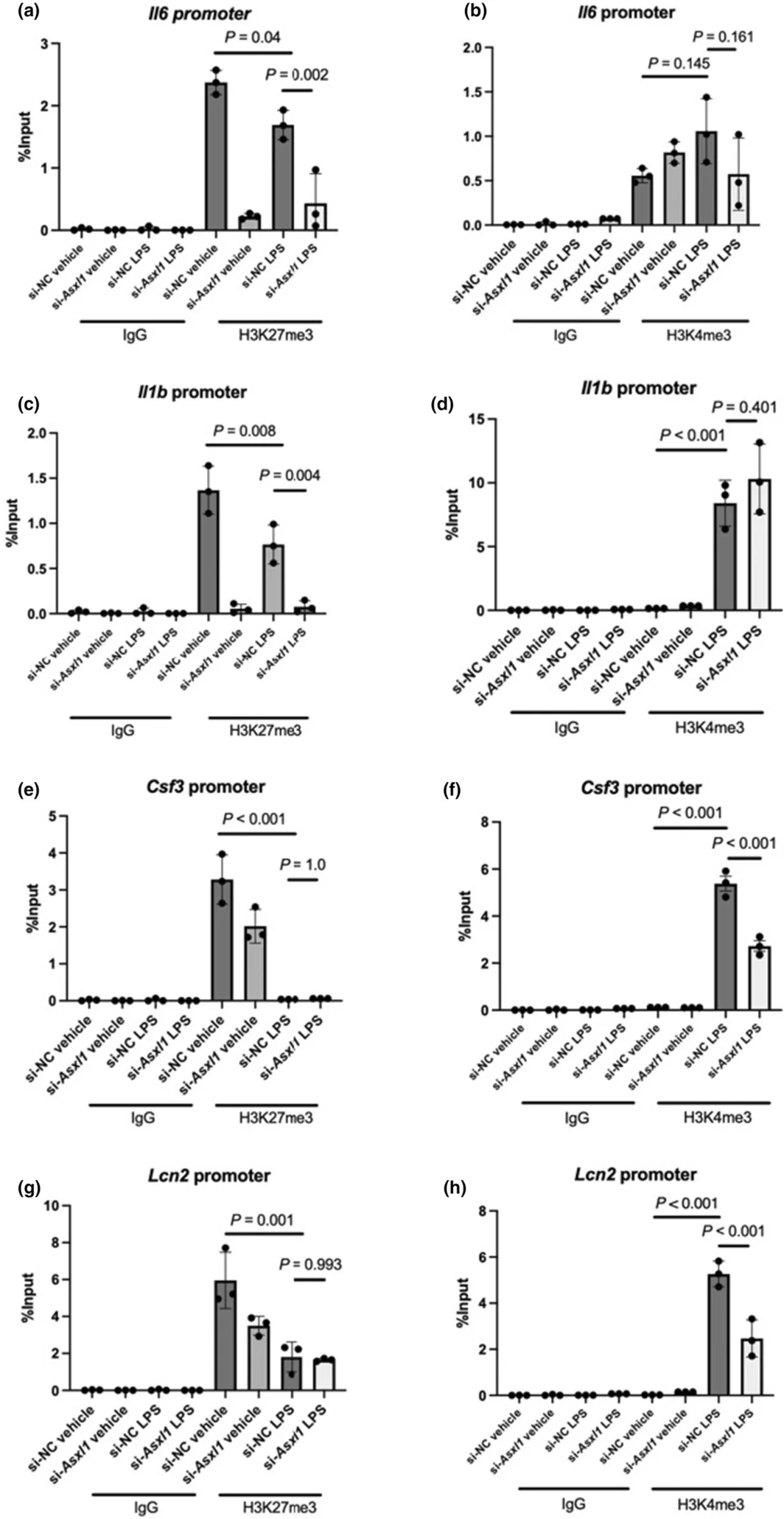
Silencing *Asxl1* suppresses mainly H3K27me3 levels at the *Il6* and *Il1b* promoters; in addition, it suppresses mainly H3K4me3 levels at the *Csf3* and *Lcn2* promoters in M1 macrophages. (*A*‐*H*) Chromatin immunoprecipitation analysis of H3K27me3 and H3K4me3 levels at the *Il6* promoter (a, b), the *Il1b* promoter (c, d), the *Csf3* promoter (e, f), and the *Lcn2* promoter (g, h) in si‐NC or si‐*Asxl1* RAW 264.7 cells treated with vehicle or LPS. Normal rabbit or mouse IgG was used as a negative control. Comparisons among four groups were made using one‐way analysis of variance followed by Dunnett's multiple comparisons post hoc test. The above data represent three independent repeated experiments, and the data are presented as mean ± standard deviation; *n* = 3. IgG, immunoglobulin G; NC, negative control; LPS, lipopolysaccharide.

Next, we found that H3K4me3 levels were markedly increased at anti‐apoptotic genes (*Csf3* and *Lcn2*) promoters in LPS‐stimulated macrophages compared with vehicle‐treated cells (both *p* < 0.001). Silencing *Asxl1* significantly reduced H3K4me3 enrichment at these promoters to 0.51 ± 0.06‐fold (*p* < 0.001) and 0.47 ± 0.13‐fold (*p* < 0.001), respectively (Figure 7f,h), leading to decreased expression of *Csf3* and *Lcn2*. Levels of H3K27me3 were remarkably reduced at the *Csf3* and *Lcn2* promoters in LPS‐stimulated cells compared with vehicle‐treated cells (*p* < 0.001 and *p* = 0.001, respectively), and silencing *Asxl1* did not affect the level of H3K27me3 in LPS‐stimulated macrophages (*p* = 1.0 and *p* = 0.993, respectively). These results indicate that ASXL1 is influencing mainly H3K4me3 levels at the promoters of *Csf3* and *Lcn2*, and maintains H3K4me3 levels at the promoters of these anti‐apoptotic genes in M1 macrophages, thereby supporting their transcriptional activation.

We also investigated histone modifications at the promoters of other inflammatory genes (*Edn1*, *Ccl7*, and *Nos2*). The histone modifications at the *Edn1* promoter showed a similar trend to those of *Il6* and *Il1b*. Level of H3K27me3 at the promoter of *Edn1* was significantly reduced in LPS‐stimulated cells compared with vehicle‐treated cells to 0.76 ± 0.03‐fold (*p* = 0.021), and silencing *Asxl1* significantly further reduced H3K27me3 enrichment at the promoter to 0.1 ± 0.08‐fold (*p* < 0.001) (Figure S5A). In contrast, Level of H3K4me3 at the promoter of *Edn1* was significantly elevated in LPS‐stimulated cells compared with vehicle‐treated cells to 5.32 ± 0.8‐fold (*p* = 0.001), and silencing *Asxl1* did not change H3K27me3 enrichment at the promoter to 0.84 ± 0.17‐fold (*p* = 0.659) (Figure S5B). Regarding *Ccl7* promoter, level of H3K27me3 tended to be reduced in LPS‐stimulated cells compared with vehicle‐treated cells to 0.75 ± 0.16‐fold (*p* = 0.144), and silencing *Asxl1* also tended to decrease the H3K27me3 level further 0.58 ± 0.03‐fold (*p* = 0.064) (Figure S5C). In addition, H3K4me3 level was significantly increased at the *Ccl7* promoter in LPS‐stimulated macrophages compared with vehicle‐treated cells—6.78 ± 3.32‐fold (*p* = 0.024). Then, silencing *Asxl1* decrease the H3K4me3 level to 0.14 ± 0.07‐fold (*p* = 0.023) in LPS‐stimulated macrophages (Figure S5D). This result indicated that *Asxl1* is influencing mainlyH3K4me3 level at the *Ccl7* promoter. However, it was not compatible completely with the fact that silencing *Asxl1* in LPS‐stimulated macrophages enhanced *Ccl7* expression (Figure 3d). With regard to *Nos2* promoter, level of H3K27me3 was reduced in LPS‐stimulated cells compared with vehicle‐treated cells to 0.37 ± 0.13‐fold (*p* = 0.006), and silencing *Asxl1* did not change the H3K27me3 level (*p* = 0.387) (Figure S5E). Additionally, H3K4me3 level was significantly increased at the *Nos2* promoter in LPS‐stimulated macrophages compared with vehicle‐treated cells—6.22 ± 0.64‐fold (*p* < 0.001). Then, silencing *Asxl1* decrease the H3K4me3 level to 0.10 ± 0.05‐fold (*p* < 0.001) in LPS‐stimulated macrophages (Figure S5F). This finding indicates that ASXL1 is influencing mainly H3K4me3 levels at the promoters of *Nos2*, and maintains H3K4me3 levels at the promoter of *Nos2* in M1 macrophages, which is a similar trend to those of *Csf3* and *Lcn2*.

## 4 DISCUSSION

ASXL1 in macrophages has been implicated in kidney injury, including AKI, in the context of clonal hematopoiesis (Vlasschaert et al., 2022; Vlasschaert et al., 2024). A recent clinical study reported an association between *Asxl1*‐related CHIP and AKI (Vlasschaert et al., 2024). Acquired mutations in hematopoietic stem cells, which occur predominantly in the elderly, are thought to give rise to proinflammatory CHIP‐mutant macrophages that infiltrate the kidneys and contribute to tubular damage (Pan et al., 2024). However, the underlying mechanisms by which ASXL1 functions in macrophages accumulating in injured kidneys remain unclear. To our knowledge, this is the first study to elucidate the functional role of ASXL1 in inflammatory macrophages during AKI.

While ASXL1 is known to regulate essential epigenetic functions (Asada et al., 2019; Sinclair et al., 1998), its specific expression pattern during kidney injury has not been well characterized. In our in vivo models, ASXL1 expression patterns in tubular epithelial cells and PDGFRβ‐positive cells remained largely unchanged between sham and uIRI kidneys. Although bulk kidney *Asxl1* mRNA levels did not significantly differ—likely because baseline expression is predominantly tubular—we observed a distinct local response in the macrophage compartment. Specifically, flow cytometry analysis revealed that the ASXL1‐positive ratio of macrophages significantly increased in the acute phase of uIRI kidneys. Furthermore, nearly one‐quarter of M1 macrophages were ASXL1‐positive, and these cells substantially accumulated in the renal interstitium 24 h after reperfusion.

Consistent with a public single‐cell RNA‐seq dataset of mouse IRI (Kirita et al., 2020), our immunofluorescence analysis confirmed ASXL1 expression in tubular epithelial cells, PDGFRβ‐positive cells, and macrophages. Notably, ASXL1 was detected in both the nucleus and cytoplasm. Although our study focuses on its classical nuclear epigenetic role, recent studies have shown that ASXL1 also functions as a cytoplasmic signaling modulator (Fujino et al., 2021; Sato et al., 2024). Therefore, our cytoplasmic staining accurately reflects its known physiological distribution. Collectively, while the precise in vivo function of ASXL1 in these cells remains to be determined, these findings suggest that ASXL1‐positive M1 macrophages might be associated with the inflammatory processes emerging in AKI.

M1 macrophages exhibit multiple functional roles, including proinflammatory activity, high microbicidal capacity, clearance of cellular debris, initiation of tissue remodeling during the inflammatory phase, efferocytosis, and anti‐apoptotic effects (Boada‐Romero et al., 2020; Bonventre & Yang, 2011; Mass et al., 2023; Murray & Wynn, 2011; Nishida & Hamaoka, 2006; Zang et al., 2014). Activated macrophages release large quantities of inflammatory cytokines that promote necrosis or apoptosis of tubular cells while simultaneously secreting anti‐apoptotic factors that facilitate the resolution of inflammation. Epigenetic mechanisms have increasingly been recognized as key regulators of macrophage phenotype and functional plasticity (Gosselin & Glass, 2014; Kittan et al., 2013). In vitro study, we found that *Asxl1* acts as a negative regulator of IL‐6 production in LPS‐stimulated RAW 264.7 cells; therefore, histone modification regulated by *Asxl1* in M1 macrophages was considered noteworthy.

H3K27me3 is a histone modification associated with gene‐specific regulation in innate immunity (Li et al., 2017; Lin et al., 2023). ASXL1 interacts with core components of PRC2, including EZH2, SUZ12, and EED, facilitating H3K27 trimethylation (Abdel‐Wahab et al., 2012). Depletion of ASXL1 has been shown to cause a genome‐wide global reduction in H3K27me3 levels in hematopoietic cells (Abdel‐Wahab et al., 2012). In the present study, RNA sequencing revealed that silencing *Asxl1* enhanced inflammatory response pathways and increased the expression of inflammatory cytokines such as IL‐6, IL‐1β, EDN1, and CCL7, thereby promoting M1 macrophage polarization. Chromatin immunoprecipitation analysis at the promoters of these genes further demonstrated that the main histone modification regulated by ASXL1 was not H3K4me3 but H3K27me3 in M1 macrophages, at least for *Il6*, *Il1b*, and *Edn1*. In addition, TRRUST analysis showed NF‐κB is the major upstream regulator of the inflammatory response pathways. These findings suggest that loss of ASXL1 impairs PRC2 function, resulting in reduced H3K27me3 levels at inflammatory cytokine gene promoters. This reduction may enhance the accessibility of transcription factors such as NF‐κB, leading to increased transcription of inflammatory cytokines. Collectively, these results indicate that ASXL1 acts as a negative regulator of inflammatory cytokine overactivation and modulates M1 macrophage polarization by maintaining H3K27me3‐mediated transcriptional repression, possibly involving NF‐κB‐related transcriptional networks.

In addition to its role in the Polycomb repressive complex, ASXL1 also functions as part of the TrxG to promote H3K4 trimethylation (H3K4me3). ASXL1 has been reported to collaborate with other TrxG components to induce H3K4me3, and *Asxl1* knockdown has been shown to reduce global H3K4me3 levels in human leukemic cells (Inoue et al., 2018). In our RNA sequencing analysis, the IL‐17 signaling pathway was suppressed following *Asxl1* silencing in M1 macrophages. The IL‐17 cytokine family (IL‐17A–F) produced by T helper 17 cells plays essential roles in host defense against microbial pathogens through the crosstalk with other immune cells including macrophages, CD4 T cells, and B cells (Gu et al., 2013; McAleer & Kolls, 2011). In addition, macrophages express IL‐17 receptors, and IL‐17 was reported to regulate the antimicrobial effect of M1 macrophages (Andrade et al., 2025), hence antimicrobial and host defense ability was estimated to be impaired in M1 macrophages whose *Asxl1* was knocked down in relation to the IL‐17 signaling pathway. Among the genes associated with this pathway, *Csf3* and *Lcn2*, which encode anti‐apoptotic and renoprotective factors (Chang et al., 2015; Ondee et al., 2019), were among the most downregulated differentially expressed genes that were upregulated by LPS stimulation but suppressed by *Asxl1* knockdown. Chromatin immunoprecipitation analysis at the *Csf3* and *Lcn2* promoters further demonstrated that the main histone modification regulated by ASXL1 was not H3K27me3 but H3K4me3 in M1 macrophages. *Nos2* expression was also suppressed with the reduction of H3K4me3 levels at the *Nos2* promoter by silencing Asxl1 in M1 macrophages. In addition, TRRUST analysis showed NF‐κB is the major upstream regulator of inflammatory and survival‐related pathways. These findings suggest that loss of ASXL1 impairs TrxG‐associated function, leading to decreased H3K4me3 levels at the promoters of *Csf3*, *Lcn2*, and *Nos2*. The resulting reduction in chromatin accessibility likely limits transcription factor binding, including that of NF‐κB, thereby suppressing gene transcription. Consequently, *Asxl1* silencing diminishes the anti‐apoptotic capacity of M1 macrophages and may exacerbate tubular injury in AKI. Besides, *Nos2* is known to generate nitric oxide (NO) in M1 macrophages and contributes to the killing of intracellular microbial pathogens (Nathan & Shiloh, 2000). ASXL1 thus appears to preserve the anti‐apoptotic and microbicidal functions of M1 macrophages by maintaining H3K4me3 marks at the promoters of *Csf3*, *Lcn2*, and *Nos2*.

Silencing *Asxl1* in M1 macrophages led to a reduction in the phagocytosis ratio, suggesting that ASXL1 contributes to maintaining the phagocytic capacity of these cells. Phagocytosis of dying cells by macrophages, known as efferocytosis, plays a crucial role in the resolution phase of inflammation (Kourtzelis et al., 2020). RNA sequencing analysis revealed that the FCGR‐dependent phagocytosis pathway was downregulated upon *Asxl1* knockdown. Within this pathway, the *Fcgr2b* gene—encoding the inhibitory receptor FCGR IIb—was identified. The inhibitory function of FCGR IIb is essential for maintaining immune balance and has been implicated in several autoimmune diseases (Bakema & van Egmond, 2014; Bournazos et al., 2020; Espeli et al., 2016). Furthermore, macrophage‐specific FcγRIIB deficiency has been reported to enhance activation of the NLR family pyrin domain containing 3 (NLRP3) inflammasome through the TLR4/MyD88 signaling pathway in a mouse model of IgA nephropathy (Liu et al., 2025). Taken together, these findings suggest that *Asxl1* may regulate inflammatory responses by modulating macrophage phagocytic capacity and transcriptional control of *Fcgr2b*.

In the co‐culture experiment, the supernatant from si‐*Asxl1* M1 macrophages induced more pronounced apoptosis and greater suppression of tubular epithelial cell viability than that from si‐NC M1 macrophages. Silencing *Asxl1* enhanced the expression of inflammatory cytokines while reducing the expression of anti‐apoptotic factors by decreasing H3K27me3 and H3K4me3 levels, respectively. Moreover, IL‐6 is known to influence not only apoptosis but also cell proliferation and survival (Groza et al., 2022), while LCN2 has been reported to inhibit apoptosis and promote tubular cell proliferation in ischemia–reperfusion injury models (Mori et al., 2005; Zang et al., 2014). Therefore, the reduced proliferation of tubular epithelial cells observed in the co‐culture experiment following *Asxl1* silencing in M1 macrophages may be partially attributed to increased IL‐6 levels and decreased LCN2 expression.

In this study, we revealed the elevation of ASXL1 expression in M1 macrophages in uIRI kidneys and elucidated the role of ASXL1 in M1 macrophages, demonstrating that it suppresses excessive inflammatory cytokine expression and preserves anti‐apoptotic activity through the regulation of histone modifications at H3K27me3 and H3K4me3 in vitro. From the in vivo and in vitro study, we assumed that the elevation of ASXL1 expression in M1 macrophages in uIRI kidneys is due to the compensatory mechanism for protecting kidneys from excessive inflammation. However, to date, the upstream factors that regulate ASXL1 expression in M1 macrophages have not been identified. Currently, the clinical management of AKI relies heavily on supportive care. Our findings raise the possibility that preserving ASXL1 function in proinflammatory macrophages may help limit macrophage‐mediated tubular injury. Therefore, developing pharmacologic agents capable of upregulating ASXL1 expression in macrophages could offer a novel, targeted approach for both the prevention and treatment of AKI in clinical practice. Further studies are warranted to identify and characterize the mechanisms and molecules that enhance ASXL1 expression in macrophages.

There remain several questions for future exploration. First, the difference in the inflammatory phenotype between ASXL1‐positive and ‐negative M1 macrophages in uIRI kidneys at AKI phase remains unclear. Analysis of the inflammatory response in addition to the evaluation of histone modification between the two groups is required. Second, although we found that ASXL1 targets both H3K27me3 and H3K4me3 levels at the promoters of inflammatory and anti‐apoptotic genes, how these are balanced is still open to debate. Regarding *Ccl7*, there was a discrepancy between the mRNA expression and the status of histone modification in LPS‐stimulated macrophages following *Asxl1* silencing. Evaluating histone modifications alone is insufficient; hence further detailed investigation of the binding of transcription factors such as NF‐κB by ChIP is also needed. Third, while we demonstrated that ASXL1 is expressed in tubular epithelial cells and fibroblasts in addition to macrophages in the kidneys, their specific functions remain unknown and warrant further examination. Fourth, all in vivo experiments in this study were conducted exclusively using male mice. Given that sex differences are known to significantly influence inflammatory responses and susceptibility in kidney diseases (Klein & Flanagan, 2016; Neugarten & Golestaneh, 2022), it remains unclear whether similar ASXL1‐mediated macrophage regulation occurs in female mice or female patients. Future studies incorporating both sexes are essential to confirm the broader applicability of our findings.

In conclusion, ASXL1 suppresses excessive inflammatory cytokine expression and preserves the anti‐apoptotic function of M1 macrophages through histone modification. In addition, ASXL1 is involved in maintaining the phagocytic capacity of M1 macrophages. Overall, these findings suggest that ASXL1 in M1 macrophages may epigenetically limit macrophage‐mediated renal tubular epithelial cell damage by attenuating inflammatory responses and maintaining anti‐apoptotic activity and phagocytosis.

## AUTHOR CONTRIBUTIONS

**Yoshiyasu Ogura:** Conceptualization; data curation; formal analysis; investigation; methodology; project administration; validation; visualization. **Takehito Sugasawa:** Data curation; formal analysis; resources; software; visualization. **Masaomi Nangaku:** Conceptualization; project administration; resources; supervision. **Imari Mimura:** Conceptualization; data curation; formal analysis; funding acquisition; investigation; methodology; project administration; resources; supervision; validation; visualization.

## FUNDING INFORMATION

This study was in part supported by the Grant‐in‐Aid for Scientific Research (C) 20K08604 and 25K11537 (I.M.) by the Japan Society for the Promotion of Science (JSPS) KAKENHI. This study was also supported by the Takeda Science Foundation (I.M.), Koyanagi Foundation (I.M.), Chugai Foundation for Innovative Drug Discovery Science (I.M.) and Mitsui Sumitomo Insurance Welfare Foundation (I.M.).

## CONFLICT OF INTEREST STATEMENT

The authors declare no conflicts of interest.

## Supporting information


**Figure S1.** Gating strategy for flow cytometric analysis of dissociated kidney cells. After the doublet discrimination, Live leukocytes were identified by CD45 expression, Live/dead marker exclusion and scatter properties. Among total macrophages (Ly‐6G‐, CD11b+, and F4/80+ cells), subsets were defined according to ASXL1 and CD86 (M1 marker).
**Figure S2.** (A) Representative images of immunofluorescence for ASXL1 (green) of testis. Hoechst (blue) was used as a nucleus stain. (B) Representative images of immunofluorescence for ASXL1 (green) and KIM1 (red). Hoechst (blue) was used as a nucleus stain. ASXL1 and KIM1 double positive tubular epithelial cells appeared in uIRI kidneys. (*C*) Representative images of immunofluorescence for ASXL1 (green) and PDGFRβ (red). Hoechst (blue) was used as a nucleus stain. ASXL1 and PDGFRβ double positive cells (white arrowheads) were increased in uIRI kidneys. Bar = 50 μm, Original magnification: ×400.
**Figure S3.** Heatmap of the 229 gene upregulated by both LPS stimulation and Asxl1 silencing in the transcriptome analysis of RAW 264.7 cells.
**Figure S4.** Heatmap of the 283 genes upregulated by LPS stimulation and suppressed by Asxl1 silencing in the transcriptome analysis of RAW 264.7 cells.
**Figure S5.** Chromatin immunoprecipitation analysis of H3K27me3 and H3K4me3 levels at the *Edn1* promoter (A, B), *Ccl7* promoter (C, D) and the *Nos2* promoter (E, F) in si‐NC or si‐Asxl1 RAW 264.7 cells treated with vehicle or LPS. Normal rabbit or mouse IgG was used as a negative control. Comparisons among four groups were made using one‐way analysis of variance followed by Dunnett's multiple comparisons post hoc test. The above data represent three independent repeated experiments, and the Data are presented as mean ± standard deviation; *n* = 3. IgG, immunoglobulin G; NC, negative control; LPS, lipopolysaccharide.

## Data Availability

All the data and materials supporting the findings of this study are available within the article. Further requests can be directed by email to the corresponding author.
