# Supplementary material for: ASXL1 in proinflammatory macrophages attenuates inflammatory activation and macrophage‐mediated renal tubular epithelial cell damage in acute kidney injury
Source: Physiol Rep. 2026 Sep 30;14(19):e71119. doi: 10.14814/phy2.71119 (PMC13749610; doi:10.14814/phy2.71119)
Supplement: Supplementary file 1 [file PHY2-14-e71119-s001.pdf]

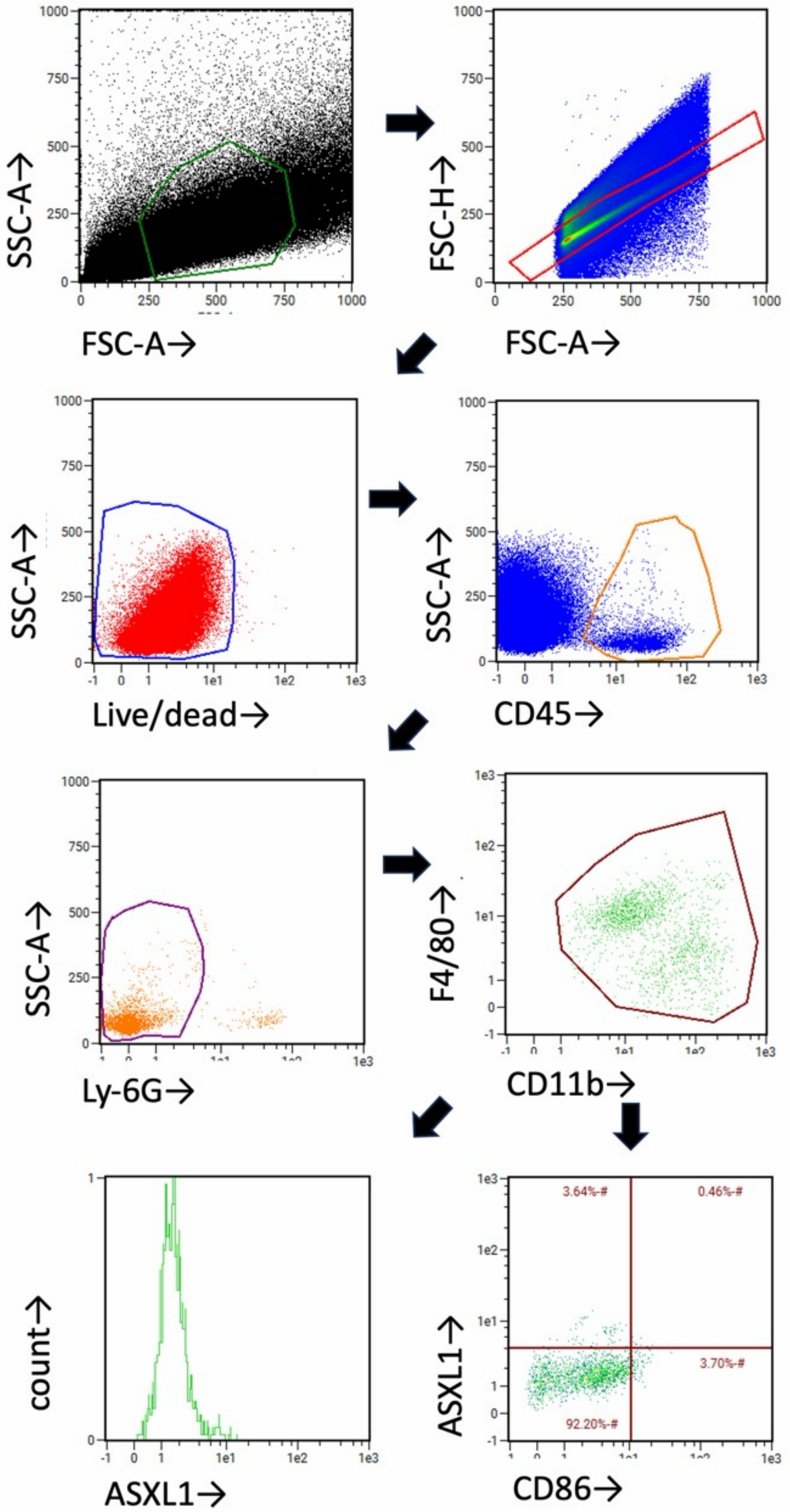

Supplemental Figure 1. Gating strategy for flow cytometric analysis of dissociated kidney cells. After the doublet discrimination, Live leukocytes were identified by CD45 expression, Live/dead marker exclusion and scatter properties. Among total macrophages (Ly-6G$^-$, CD11b$^+$ and F4/80$^+$ cells), subsets were defined according to ASXL1 and CD86 (M1 marker).

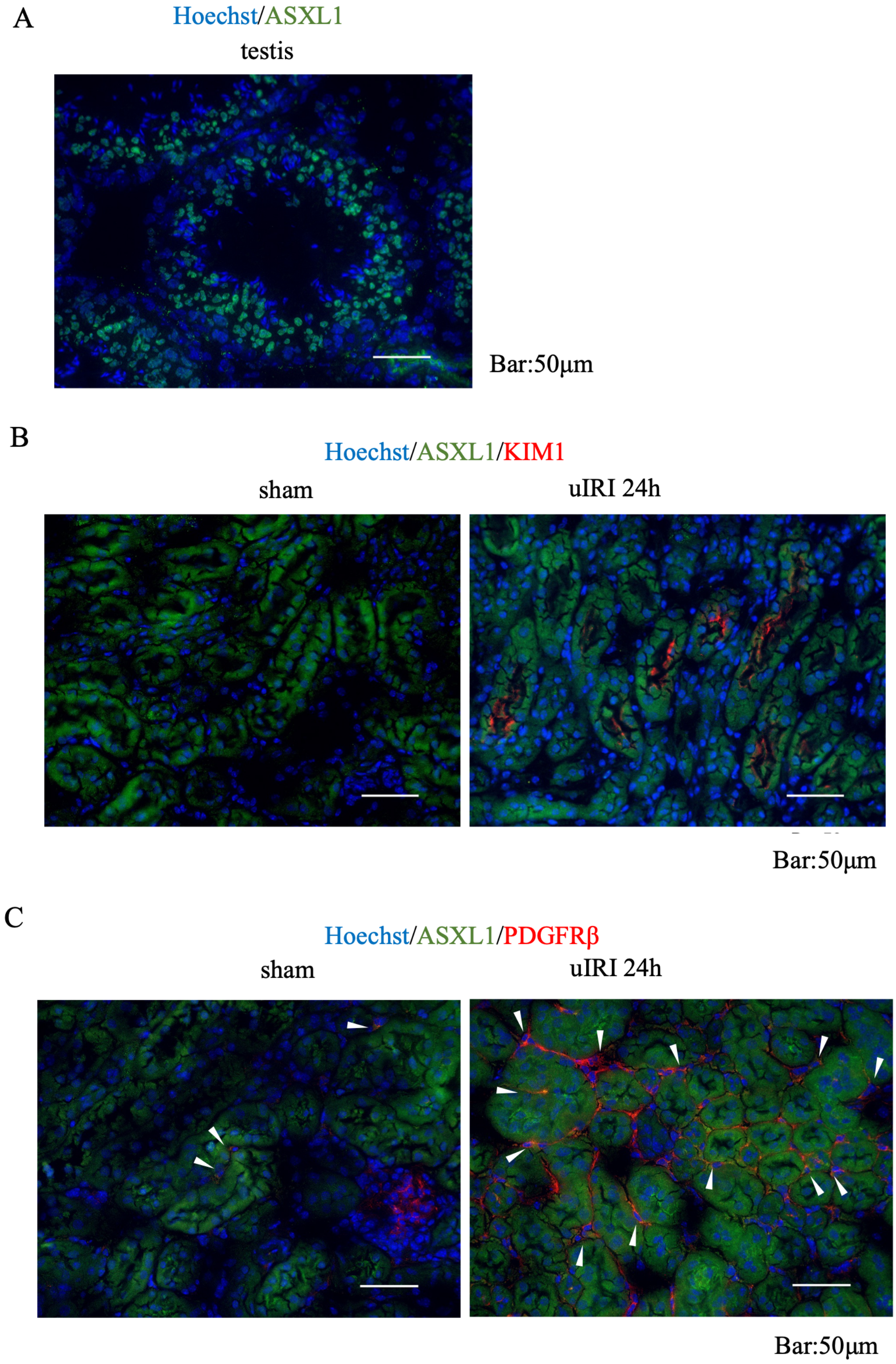

Supplemental Figure 2. (*A*) Representative images of immunofluorescence for ASXL1 (green) of testis. Hoechst (blue) was used as a nucleus stain. (*B*) Representative images of immunofluorescence for ASXL1 (green) and KIM1 (red). Hoechst (blue) was used as a nucleus stain. ASXL1 and KIM1 double positive tubular epithelial cells appeared in uIRI kidneys. (*C*) Representative images of immunofluorescence for ASXL1 (green) and PDGFRβ (red). Hoechst (blue) was used as a nucleus stain. ASXL1 and PDGFRβ double positive cells (white arrowheads) were increased in uIRI kidneys. Bar=50µm, Original magnification: ×400.

Supplemental Figure 3. Heatmap of the 229 gene upregulated by both LPS stimulation and Asxl1 silencing in the transcriptome analysis of RAW 264.7 cells.

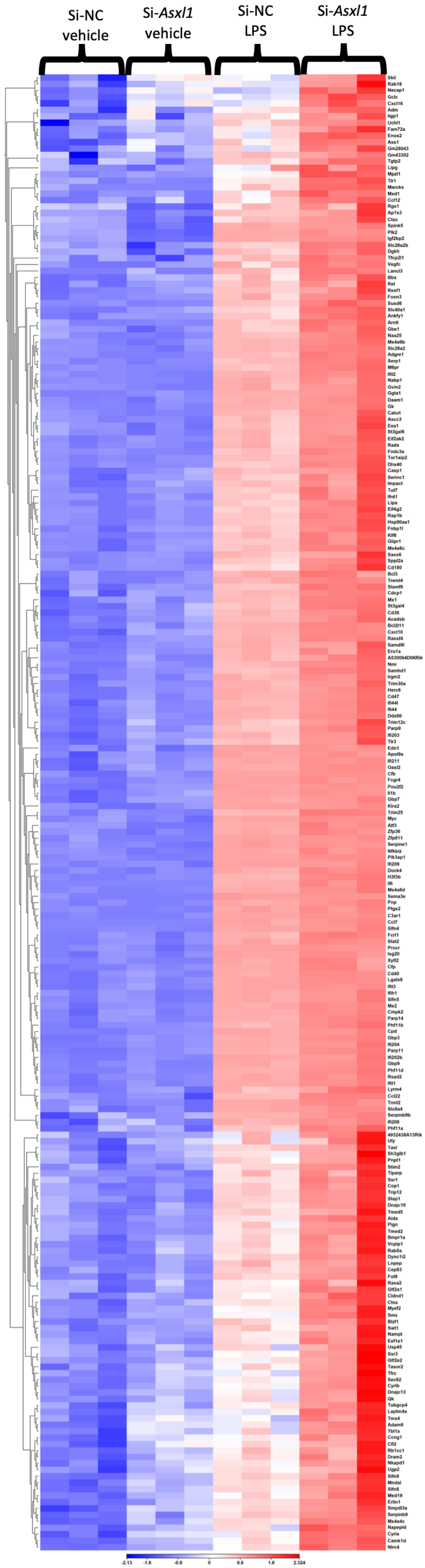

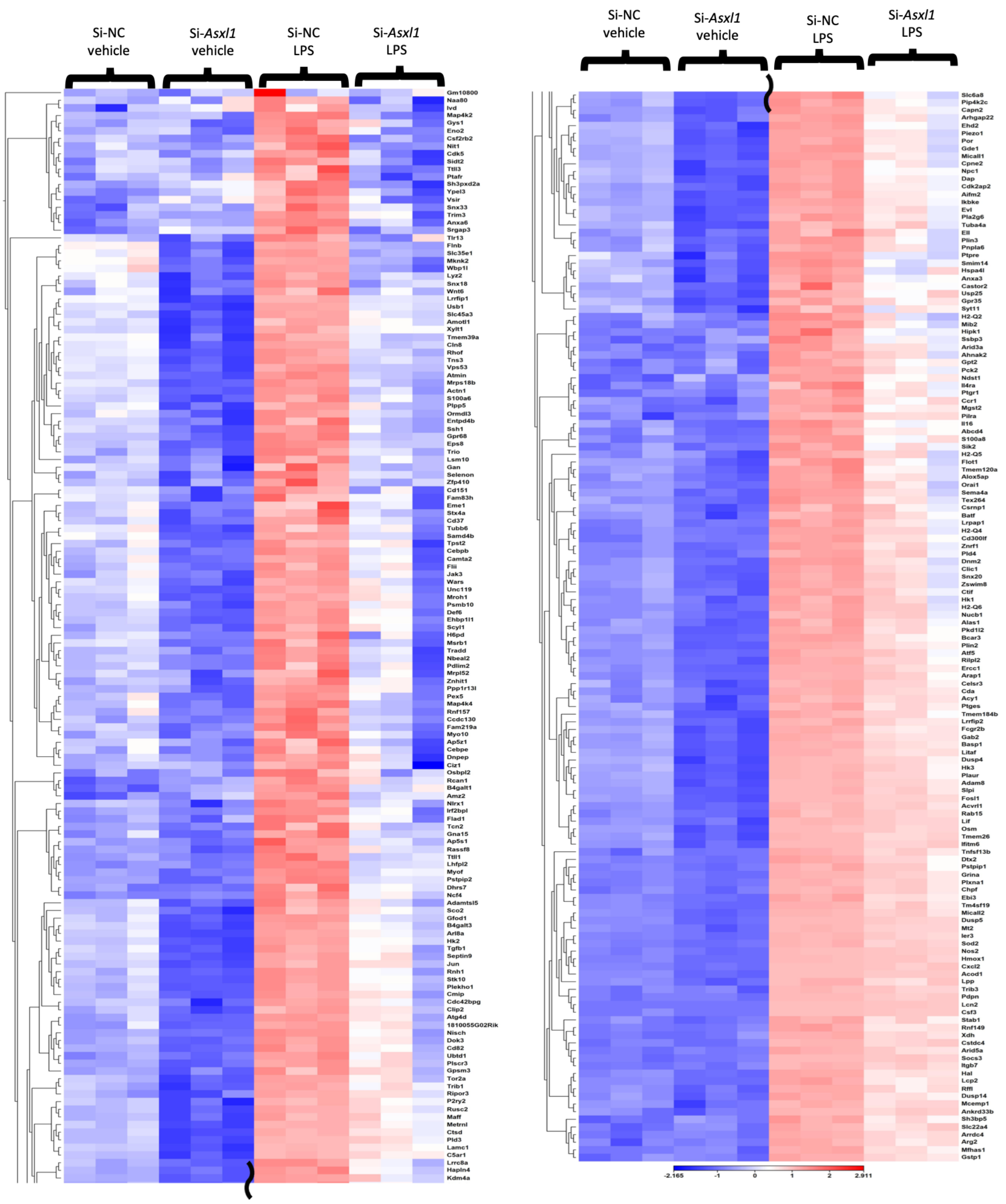

Supplemental Figure 4. Heatmap of the 283 genes upregulated by LPS stimulation and suppressed by Asxl1 silencing in the transcriptome analysis of RAW 264.7 cells.

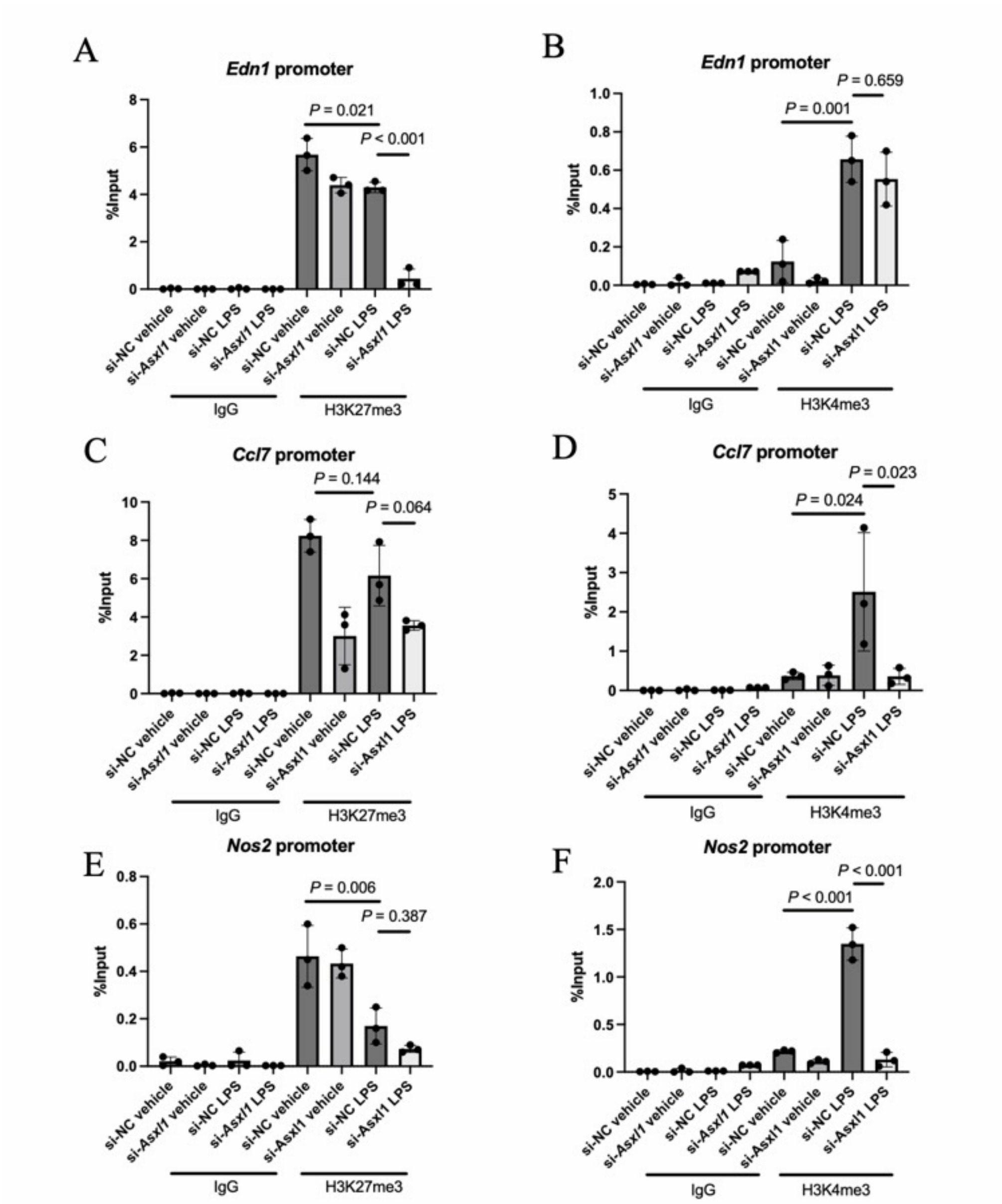

Supplemental Figure 5. Chromatin immunoprecipitation analysis of H3K27me3 and H3K4me3 levels at the *Edn1* promoter (*A*, *B*), *Ccl7* promoter (*C*, *D*) and the *Nos2* promoter (*E*, *F*) in si-NC or si-Asxl1 RAW 264.7 cells treated with vehicle or LPS.

Normal rabbit or mouse IgG was used as a negative control. Comparisons among four groups were made using one-way analysis of variance followed by Dunnett's multiple comparisons post hoc test. The above data represent three independent repeated experiments, and the Data are presented as mean ± standard deviation; n = 3.

IgG, immunoglobulin G; NC, negative control; LPS, lipopolysaccharide.
